# Systematic Pan-Cancer Analysis Identifies CDK1 as an Immunological and Prognostic Biomarker

**DOI:** 10.1155/2022/8115474

**Published:** 2022-08-31

**Authors:** Yaqi Yang, Qin Liu, Xiyuan Guo, Qing Yuan, Siji Nian, Pengyuan Kang, Zixi Xu, Lin Li, Yingchun Ye

**Affiliations:** Immune Mechanism and Therapy of Major Diseases of Luzhou Key Laboratory, Public Center of Experimental Technology, School of Basic Medical Sciences, Southwest Medical University, Luzhou 646000, China

## Abstract

Cyclin-dependent kinase 1 (CDK1) plays an important role in cancer development, progression, and the overall process of tumorigenesis. However, no pan-cancer analysis has been reported for CDK1, and the predictive role of CDK1 in immune checkpoint inhibitors (ICIs) therapy response remains unexplored. Thus, in this study, we first investigated the potential oncogenic role of CDK1 in 33 tumors by multidimensional bioinformatics analysis based on The Cancer Genome Atlas (TCGA) and Gene Expression Omnibus (GEO) datasets. Bioinformatic analysis and immunohistochemical experiments confirmed that CDK1 is significantly upregulated in most common cancers and is strongly associated with prognosis. Further analysis indicated that CDK1 may influence tumor immunity mainly by mediating the degree of tumor infiltration of immune-associated cells, and the effect of CDK1 on immunity is diverse across tumor types in tumor microenvironment. CDK1 was also positively correlated with tumor mutational burden (TMB) and microsatellite instability (MSI) in certain cancer types, linking its expression to the assessment of possible treatment response. The results of the pan-cancer analysis study showed that the CDK1 gene was positively associated with the expression of three classes of RNA methylation regulatory proteins, and affects RNA function through multiple mechanisms of action and plays an important role in the posttranscriptional regulation of the tumor microenvironment. These findings shed light on the role of the CDK1 gene in cancer progression and provide information to further study the CDK1 gene as a potential target for pan-cancer.

## 1. Introduction

Cancer is the leading cause of human mortality worldwide and a significant barrier to a long life. The health and economic burdens associated with its high incidence and mortality are rapidly increasing [1, 2]. Cancer is a malignant disease and is caused by a heterogeneous population of cells with different tumorigenic abilities, phenotypes, and functions. Dysregulation of the cell cycle resulting in uncontrolled cell proliferation and genomic and chromosomal instability are common in human cancers [3–5]. Cyclins and cyclin-dependent kinases (CDKs) are the central components of the cell cycle regulatory machinery [6].

CDKs belong to the serine/threonine-specific protein kinase family, which are essential for normal cell cycle progression and are the key regulatory enzymes that drive all cell cycle transitions and coordinate the progression of the entire cell cycle in all eukaryotic cells [6–8]. Additionally, CDKs are also involved in gene transcription, mRNA processing, and cell differentiation [9, 10]. Dysregulation of CDKs leads to the sustained or spontaneous proliferation of tumor cells and accelerates the malignant growth of tumors. To date, 21 CDK and 5 CDK-like genes have been identified in the human genome based on their homologous sequences [11]. Among them, CDK1, also known as cell division cycle 2 (CDC2), is the only essential CDK in human cells. CDK1 can replace other CDKs and effectively drive the mammalian cell cycle, controlling the transition of cells from the G2 phase to the M phase [12–14]. Moreover, in malignant tumor cells, the altered expression of CDK1 and its regulators can lead to uncontrolled CDK1 activity, which can cause uncontrolled proliferation of tumor cells and aggravate the malignancy of the tumor. Hence, CDK1 is a potential target for tumor therapy.

In advanced gastrointestinal stromal tumors [15], bladder cancer [16], non-small cell lung cancer [17], and melanoma [18], high CDK1 expression promotes the progression of malignant tumors and exacerbates the degree of proliferation of malignant tumor cells. However, current CDK1-based studies are limited to a single tumor type, and the role of the CDK1 gene in human cancers and the overall CDK1 tumor landscape are still unknown. Thus, its relationship with human cancers needs further investigation. In recent years, pan-cancer analysis has been widely used in cancer research, providing unique, detailed, and comprehensive insights into human cancers to improve the quality of cancer analysis [19–21]. Given the complexity of tumorigenesis, it is extremely important to analyze the CDK1 gene for pan-cancer expression and to assess its relationship with clinical prognosis and its relevance to the underlying molecular mechanisms.

In this study, a pan-cancer analysis of CDK1 was performed using the TCGA project and the GEO database to explore the potential molecular mechanisms of the action of CDK1 in tumorigenesis and clinical prognosis in terms of gene expression, survival status, genetic alterations, protein phosphorylation, methylation levels, immune infiltration, pan-cancer correlation with three classes of RNA methylation regulatory proteins and related cellular pathways, and to lay the foundation for future research on CDK1-based antitumor therapy.

## 2. Materials and Methods

### 2.1. Ethical Statement

This study was approved by the clinical trial ethics committee of the Affiliated Hospital of Southwest Medical University, China (ethics review number: KY2019276). Following the approval of the ethics committee, the volunteers signed the informed consent before samples were collected, and all methods were performed as per the relevant guidelines and regulations. This study was compliant with the Declaration of Helsinki.

### 2.2. Gene Expression Analysis

Oncomine, the classic sample database in oncology, is the largest oncogene chip database and integrated data-mining platform. It contains 86,733 samples and 715 gene expression datasets, which can be accessed to mine information related to cancer genes and can assist in screening tumor-related target molecules or predicting phenotypes [22]. Therefore, we used this online database (https://www.oncomine.org) to assess the mRNA expression levels of CDK1 in different tumors. In this study, *P* values lesser than 0.01 in the Oncomine database, a fold change of 2, and a gene ranking at 10% were set as the thresholds of significance. Additionally, we used the “Gene_DE” module of the TIMER2 [23] web server (https://timer.cistrome.org/) to determine the differences in CDK1 expression between cancer tissue and adjacent normal tissues in TCGA tumors. We downloaded RNA-seq sequencing data from the TCGA and Genotype-Tissue Expression (GTEx) datasets from the UCSC XENA portal (https://xenabrowser.net/datapages/). The downloaded data were uniformly processed by the Toil process [24] and log2 (TPM+1) transformed for analysis to compare the CDK1 gene expression between tumor and normal tissue in multiple types of tumor.

UALCAN (https://ualcan.path.uab.edu) is a comprehensive, interactive web portal for a detailed analysis of the TCGA gene expression data [25]. We performed expression analysis on the Clinical Proteomic Tumor Analysis Consortium (CPTAC) dataset through this interactive web resource. The expression levels of CDK1 (NP_001307847.1) in total or phosphorylated proteins in primary tumors and normal tissues were explored. Valid datasets were selected from six tumors, including breast cancer, ovarian cancer, colon cancer, renal clear cell carcinoma (RCC), uterine corpus endometrial carcinoma (UCEC), and lung adenocarcinoma (LUAD). A violin plot of CDK1 expression in all TCGA tumors at different pathological stages (stages I, II, III, and IV) was obtained using the “Pathological stage map” module of the GEPIA2 web server (https://gepia2.cancer-pku.cn/#index) [26]. The log2 (TPM+1)-transformed expression data were visualized and analyzed in the form of violin plots to determine the expression of CDK1 in different pathological stages of different tumors.

### 2.3. Survival Prognosis Analysis

To elucidate the relationship between CDK1 regulatory genes and tumor survival prognosis, we used the “survival map” module of GEPIA2 [26] to obtain overall survival (OS) and disease-free survival (DFS) significance map data of CDK1 in all TCGA tumors. High and low cut-off (>50% or <50%, respectively) values were used as the expression thresholds for creating high-expression and low-expression cohorts. Hypothesis testing was performed using the log-rank test, and survival curves were obtained using the “survival analysis” module of GEPIA2. We used the online survival analysis tool Kaplan-Meier plotter (https://kmplot.com/analysis/) [27] to analyze the 5-year survival of patients with different tumor types using publicly available pan-cancerRNA-seq datasets to determine the effect of CDK1 gene mRNA expression levels on the OS (*n* = 7,462) and recurrence-free survival (RFS) (*n* = 4,420) of the patients.

The PronoScan database was used to determine the relationship between gene expression and patient clinical prognosis through a large collection of publicly available cancer chip datasets [28]. We used the web server of this database (https://www.abren.net/PrognoScan/) to analyze the relationship between CDK1 expression in different types of cancer and survival, such as disease-specific survival (DSS), RFS, distant recurrence-free survival (DRFS), DFS, metastasis-free survival (MFS), and distant metastasis-free survival (DMFS). The threshold was adjusted to a Cox *P* value <0.05.

### 2.4. Gene Mutation Analysis

The cBioPortal database contains the genomic characteristics of tumors at the DNA level. It provides visual and multidimensional genomic data that can be used to study mechanism of tumorigenesis [29]. We performed a pan-cancer mapping study of CDK1 through the online web platform of this database (https://www.cbioportal.org/), looking at the results of mutation frequency, mutation type, and copy number alteration (CNA) in all TCGA tumors. The information on CDK1 mutation sites can be shown in protein structure schematics or 3D structures through the “Mutations” module of this database. Also, we analyzed the correlation between CDK1 gene expression and TMB and MSI in 33 tumor types using Spearman's correlation. Paired mRNA expression data for 33 tumor types (*n* = 10,201) were downloaded from the genomic data commons (GDC) data portal (https://portal.gdc.cancer.gov/) and statistically analyzed using the *R* package (v4.0.3); *P* < 0.05 was considered statistically significant.

### 2.5. Immune Infiltration Analysis

The relationship between CDK1 expression and immune infiltration in all TCGA tumors was determined using the “Immune Gene” module of the TIMER2 database web server. Tumor-associated fibroblasts, CD8+ T cells, CD4+ T cells, B cells, dendritic cells (DC), and macrophages were selected for immune infiltration assessment based on different algorithms such as TIMER, CIBERSORT, CIBERSORT-ABS, QUANTISEQ, XCELL, MCPCOUNTER, and EPIC. The *P* values and partial correlation coefficients (COR) were calculated by Spearman's correlation and visualized in the form of heat maps and scatter plots.

### 2.6. Pan-Cancer Correlation Analysis of CDK1 and RNA Methylation Regulatory Proteins

Several modified ribonucleosides including 6-methyladenosine (m6A), 5-methylcytidine (m5C), and 1-methyladenosine (m1A) have recently been shown to occur in messenger (m)RNAs and to affect their biogenesis, translation, and stability. We downloaded the uniformly normalized pan-cancer dataset from the UCSC (https://xenabrowser.net/) database. We further extracted the expression data of ENSG00000170312 (CDK1) gene and 44 regulatory proteins involved three types (*m1A*, *m5C*, *m6A*) of RNA methylation modifications in each sample, and further we filtered the samples from the following sources: primary solid tumor, primary blood derived cancer—bone marrow, primary blood derived cancer—peripheral blood. We also filtered all normal samples, and further performed log2 (*x* + 0.001) transformation for each expression value. Next, we calculated the Pearson's correlation between CDK1 and three types of RNA methylation regulatory proteins. Visual analysis was carried out in the form of heat maps.

### 2.7. Methylation Analysis

Methylation of DNA and histones can alter the structure of the DNA. Moreover, epigenetic regulation of gene expression might be assessed by the methylation level of the promoters. We used the TCGA dataset from the UCLAN database to analyze the CDK1 promoter DNA methylation levels in various tumors to determine the differences in the methylation levels between tumors and normal tissues. The results are presented as a box plot; *P* < 0.05 was considered statistically significant.

### 2.8. CDK1-Related Gene Enrichment Analysis

STRING (https://string-db.org/) is a database for predicting protein interactions. Currently the STRING database (v11.0) contains information on more than 5,000 species, more than 20 million proteins, and more than 3 billion interactions. The database can be used to understand the complex regulatory networks in living organisms [30]. We first investigated the protein-protein interactions of CDK1-binding proteins using this database, with the main parameter settings, including the meaning of the network edge (“evidence”), the active interaction sources (“experiment”), the minimum interaction required score (“low confidence [0.150]”), and the maximum number of interactors to be shown (“no more than 50 interactors”). Finally, an interaction network of 50 experimentally identified CDK1-binding proteins was obtained.

We obtained the top 100 target genes associated with CDK1 from all the TCGA tumor and normal tissue datasets using the “similar gene detection” module of the GEPIA2 database. Jvenn, an interactive Venn diagram viewer [31], was used to analyze and visualize the interactions between CDK1-interacting genes and related genes. Moreover, we combined these two sets of gene data for the KEGG pathway analysis by uploading the gene list to the web server of the DAVID database (https://david.ncifcrf.gov) with selected identifiers (“OFFICIAL_GENE_SYMBOL”) and species (“*Homo sapiens*”). The Kyoto Encyclopedia of Genes and Genomes (KEGG) pathway analysis was performed to obtain the relevant KEGG data, and finally, using the “tidyr” and “ggplot2” *R* packages, the data were visualized. We also performed Gene Ontology (GO) functional enrichment analysis of the above genes using the Metascape database (https://metascape.org/). The data of BP (Biological process), CC (Cellular Component), and MF (Molecular Function) enrichment items were downloaded, and the results of the analysis were visualized in the form of chord and bubble diagrams.

### 2.9. Immunohistochemical Techniques

Immunohistochemistry was performed as described previously [32]. The cancer tissues and paraneoplastic tissues of human lung, breast, and liver cancers were fixed, dehydrated, embedded, and sectioned. The dewaxed sections were placed in hydrogen peroxide (containing 3% methanol) for 10 min at room temperature and washed with 1× PBS. The tissue sections were immersed in a 0.01 M citrate buffer solution (pH 6.0) and boiled. After cooling, the sections were washed with 1× PBS. A blocking solution (goat serum) was added dropwise for 20 min at room temperature. The CDK1 experimental group was incubated with recombinant anti-CDK1 rabbit monoclonal antibody (Abcam, Cat No. ab133327, Cambridge, UK) as a primary antibody, and the isotype control group was incubated with rabbit IgG1 antibody (Shanghaiyuanye Biotechnology, Cat No. S25766, Shanghai, China) overnight at 4°C. The goat anti-rabbit antibody (Zhongshan Jinqiao, Cat No. ZDR5306, Beijing, China) was added dropwise as a secondary antibody, incubated for 90 min at 37°C, and washed with 1× PBS after incubation. A DAB chromogenic reagent kit (Zhongshan Jinqiao, Cat No. K135925C, Beijing, China) was used to stain at room temperature. After hematoxylin counterstaining and dehydration, the sections were sealed with neutral gum. Semi-quantitative immunohistochemical detection was used to determine the CDK1 protein levels in different cancer tissues. The integrated optical density and corresponding area of the CDK1-positive region in the acquired immunohistochemical sections were determined using the Image-Pro Plus 6.0 image analysis system, and the mean optical density of the positive region in each immunohistochemical section was calculated. The measurements were repeated three times per section and averaged to accurately compare the difference in mean optical density between cancerous and normal tissues. (Ten cancer tissue sections and 10 normal tissue sections from patients with different tumors were stained for each cancer species, respectively. The isotype control group was also set up, and the number of normal and cancer tissue sections stained was five.)

### 2.10. Statistical Analysis

The statistical analysis was performed using Prism 8 (GraphPad Software). Data are presented as mean ± SD. Statistically significant differences between the two groups were calculated using Student's *t* test. For *P* > 0.05, the differences were considered to be statistically not significant (ns); ^*∗*^*P* < 0.05; ^*∗∗*^*P* < 0.01; and ^*∗∗∗*^*P* < 0.001.

## 3. Results

### 3.1. CDK1 Is Significantly Upregulated Expression in Most Common Cancers

**Figure 1:**
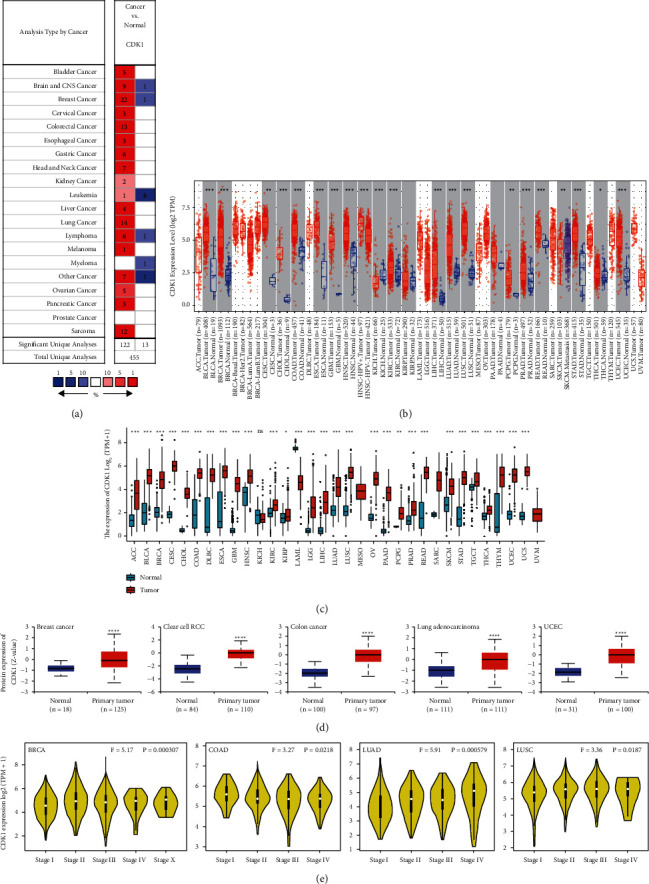
Expression levels of the CDK1 gene in different tumors and pathological stages. (a) Increase or decrease in the CDK1 levels in datasets of different cancers compared to the CDK1 levels in normal tissues in the Oncomine database. (b) CDK1 expression levels in different tumor types from the TCGA database were analyzed by TIMER2.0 (^*∗*^*P* < 0.05, ^*∗∗*^*P* < 0.01, ^*∗∗∗*^*P* < 0.001). (c) Comparison of CDK1 expression levels between tumor tissues from the TCGA database and normal tissues from the GTEx database (^*∗*^*P* < 0.05, ^*∗∗*^*P* < 0.01, ^*∗∗∗*^*P* < 0.001). (d) Based on the CPTAC dataset, the expression levels of total CDK1 protein were analyzed in normal and primary tissues of breast cancer, colon cancer, clear cell kidney cancer, and UCEC (^*∗∗∗∗*^*P* < 0.0001). (e) Correlations between CDK1 expression and tumor stage in BRCA, COAD, LUAD, and LUSC patients (Log2 (TPM + 1) was applied for log scale).

To investigate the expression of the CDK1 gene in various tumors, we analyzed the mRNA expression of the CDK1 gene using the Oncomine database (Figure 1(a)). Based on the analysis of the Oncomine database, we found that the CDK1 gene expression was higher in a variety of malignancies than in normal tissues (Figure 1(a)): these malignancies included bladder cancer, brain and CNS cancers, breast cancer, cervical cancer, colon cancer, esophageal cancer, gastric cancer, head and neck cancer, lymphoma, liver cancer, lung cancer, melanoma, ovarian cancer, and sarcoma. However, CDK1 gene expression was lower in leukemia and myeloma than in normal tissues (Figure 1(a)). We also specifically investigated the expression of the CDK1 gene in highly prevalent malignancies such as lung, gastric, liver, colon, breast, esophageal, and pancreatic cancers (Supplementary Table 1). There was a significant upregulation of CDK1 gene expression in lung cancer tissues than in normal tissues, and the results from the TCGA dataset showed a fold change of 6.939 (*P*=4.26*E* − 24) (Supplementary Table 1). The overexpression of the CDK1 gene in gastric cancer tissues showed a fold change of 2.544 (*P*=7.42*E* − 13) compared to the expression of the gene in normal tissues. Similarly, the expression levels of CDK1 gene were significantly higher in liver cancer (fold change of 5.573, *P*=1.05*E* − 84), colon cancer (fold change of 2.274, *P*=6.34*E* − 13), breast cancer (fold change of 2.325, *P*=2.36*E* − 48), esophageal cancer (fold change of 2.929, *P*=1.54*E* − 26), and pancreatic cancer (fold change of 3.888, *P*=1.37*E* − 7) than in the adjacent normal tissues (Supplementary Table 1).

To further evaluate the expression of CDK1 in human cancers, we used the TIMER2 database to analyze the expression of CDK1 in different types of TCGA tumors. The differential expression of CDK1 in all TCGA tumors versus adjacent normal tissues is shown in Figure 1(b). In bladder urothelial carcinoma (BLCA), breast invasive carcinoma (BRCA), cervical squamous cell carcinoma and endocervical adenocarcinoma (CESC), cholangiocarcinoma (CHOL), colon adenocarcinoma (COAD), esophageal carcinoma (ESCA), glioblastoma multiforme (GBM), head and neck squamous cell carcinoma (HNSC), kidney renal clear cell carcinoma (KIRC), liver hepatocellular carcinoma (LIHC), LUAD, lung squamous cell carcinoma (LUSC), pheochromocytoma and paraganglioma (PCPG), prostate adenocarcinoma (PRAD), rectum adenocarcinoma (READ), stomach adenocarcinoma (STAD), thyroid carcinoma (THCA), and UCEC, among different malignancies, the expression of CDK1 in cancer tissues was significantly higher than that in the adjacent normal tissues (all *P* values <0.05). However, in kidney chromophobe (KICH), the expression of CDK1 in cancer tissues was significantly lower than that in the adjacent normal tissues. We compared the expression levels of the CDK1 gene in the TCGA and GTEx database integrated datasets, considering the limited paracancerous normal tissue of some cancer species in the TCGA database (Figure 1(b)). The analysis showed that CDK1 expression was significantly upregulated in various tumor tissues compared to the expression in the adjacent normal tissues (Figure 1(c)).

The results of the CPTAC database showed that the expression of total CDK1 protein in breast cancer, renal clear cell carcinoma, colon cancer, lung adenocarcinoma, and UCEC tissues was higher than that in the adjacent normal tissues (*P* < 0.0001; Figure 1(d)). We then evaluated the relationship between CDK1 expression and clinicopathological staging of patients with different tumors (Figure 1(e)). The results suggested that CDK1 expression plays an important role in the clinical progression of different malignancies such as BRCA, COAD, LUAD, and LUSC, this expression pattern is associated with good clinical application prospects.

The differential overexpression of CDK1 gene in TCGA tumors lays the foundation for its potential as a tumor therapeutic target, and this differential overexpression involves more tumor types than other tumor targets, which validates its importance at the pan-cancer level.

### 3.2. High Expression of CDK1 in Tumors Significantly Reduced the Survival and Prognosis of Tumor Patients

**Figure 2:**
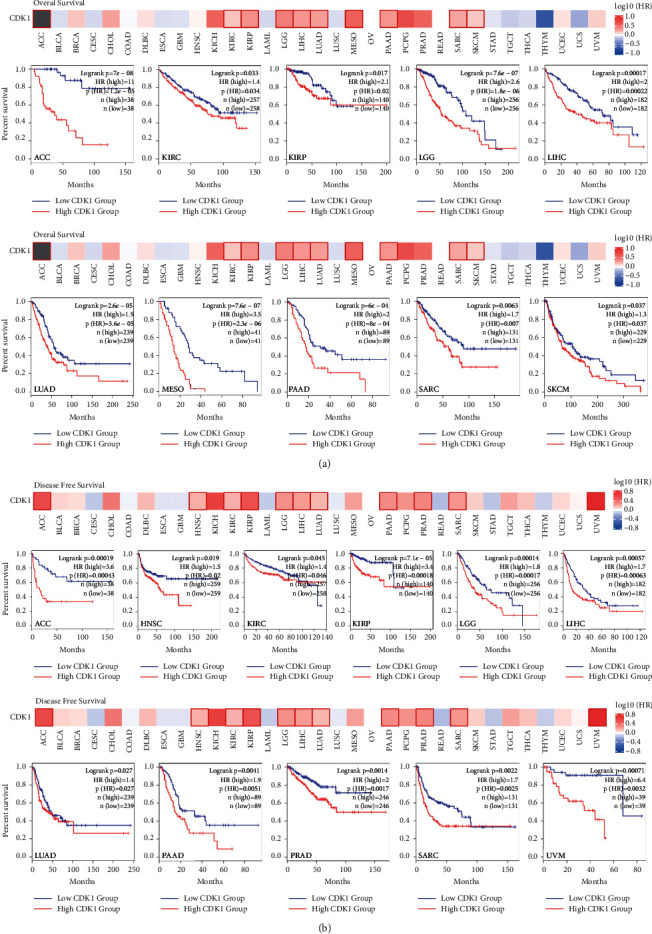
Survival analysis comparing the high and low expression of CDK1 in different types of cancer in the TCGA dataset. We used the GEPIA2 tool to perform (a) overall survival analysis and (b) disease-free survival analysis of different tumors in TCGA by CDK1 gene expression. The survival map and Kaplan-Meier curves with positive results are shown.

We divided tumor cases into high- and low-expression groups according to CDK1 expression levels and investigated the correlation between CDK1 expression and the prognosis of patients with different tumors using the TCGA dataset. As shown in Figure 2, the OS analysis data showed that high expression of CDK1 was associated with poor prognosis of TCGA tumors (Figure 2(a)). Tumor types involved adrenocortical carcinoma (ACC, *P*=7*e* − 08), KIRC (*P*=0.033), kidney renal papillary cell carcinoma (KIRP, *P*=0.017), brain lower grade glioma (LGG, *P*=76*e* − 07), LIHC (*P*=0.00017), LUAD (*P*=2.6*e* − 05), mesothelioma (MESO, *P*=7.6*e* − 07), pancreatic adenocarcinoma (PAAD, *P*=6*e* − 04), sarcoma (SARC, *P*=0.0063), and skin cutaneous melanoma (SKCM, *P*=0.037). Data from DFS analysis showed that in TCGA tumors, ACC (*P*=0.00019), HNSC (*P*=0.019), KIRC (*P*=0.045), KIRP (*P*=7.1*e* − 05), LGG (*P*=0.00014), LIHC (*P*=0.00057), LUAD (*P*=0.027), PAAD (*P*=0.0041), PRAD (*P*=0.0014), SARC (*P*=0.0022), and uveal melanoma (UVM, *P*=0.00071) and high CDK1 expression were associated with poor prognosis (Figure 2(b)).

We determined the association between CDK1 expression and prognosis of cancer patients using the Pronoscan database. Sixteen cohorts (GSE5287 [33], GSE13507 [34, 35], GSE2658 [36], GSE4475 [37], GSE12417-GPL97 [38], GSE4271-GPL96 [39], GSE4412-GPL96 [40], GSE1456-GPL96 [41], GSE12093 [42], GSE12276 [43], GSE12945 [44], GSE17537 [45], GSE9891 [46], GSE16560 [47], GSE19234 [48], and GSE30929 [49]) of the analytical data suggested that high CDK1 expression was significantly correlated with poor prognosis (Cox*P* < 0.05, Supplementary Figure 1A). The types of tumors included bladder cancer, blood cancer, brain cancer, breast cancer, colon cancer, lung cancer, ovarian cancer, prostate cancer, skin cancer, and soft tissue cancer. The impact on patient survival involved OS, DFS, DMFS, RFS, DRFS, DSS, and MFS. Notably, high expression of the CDK1 gene can be a protective factor for DFS in colorectal cancer patients. Additionally, we used the Kaplan-Meier plotter database to assess the prognostic relationship of CDK1 expression with a range of cancer types. The results showed that high expression of CDK1 significantly affected OS and RFS in tumor patients (Supplementary Figure 1B). In conclusion, these analyses consistently showed that the CDK1 gene is significantly associated with the prognosis of patients with different cancer types and can significantly influence the survival of patients with these tumors. First, we concluded that the CDK1 gene was differentially overexpressed in TCGA tumors. Meanwhile, this high expression status of CDK1 gene can significantly reduce survival time of tumor patients as revealed at the pan-cancer level. The above study lays the foundation for anti-CDK1 oncology therapy to extend the median survival time of tumor patients.

### 3.3. The Genetic and Epigenetic Features of CDK1 in Pan-Cancer

**Figure 3:**
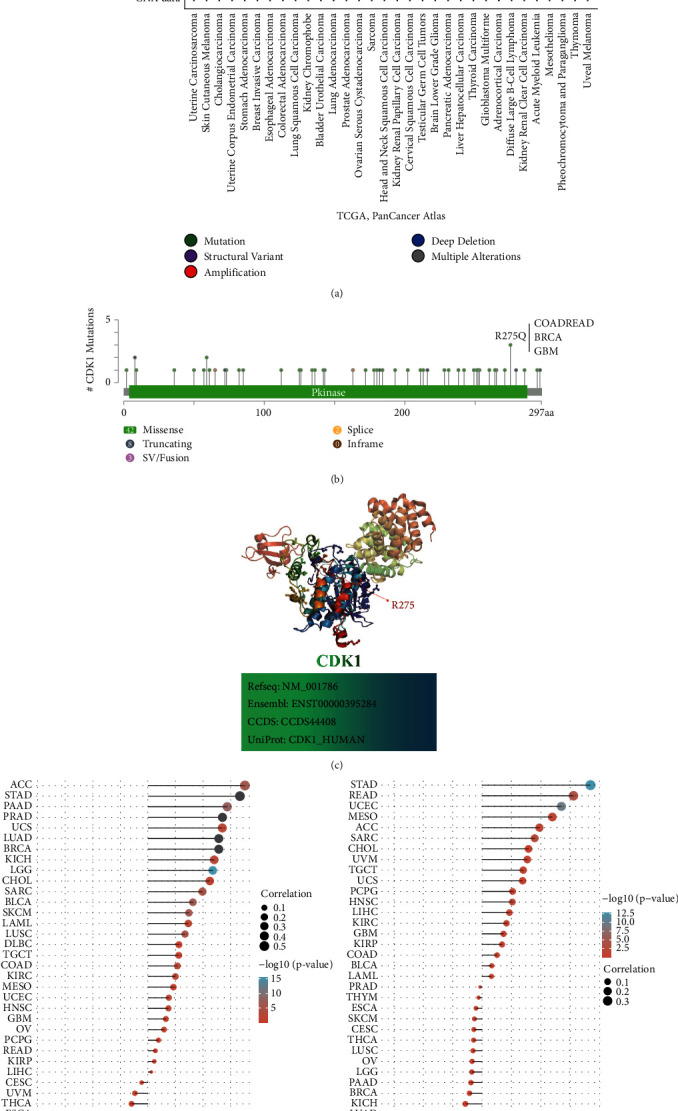
CDK1 mutation landscape. (a) The CDK1 mutation frequency in multiple TCGA pan-cancer studies according to the cBioPortal database. (b) Mutation diagram of CDK1 in different cancer types across protein domains. (c) Illustration of the three-dimensional structure of the CDK1 protein (containing the R275 mutation site). (d) Correlation between CDK1 gene expression and TCGA tumor mutation load (TMB). (e) Correlation between CDK1 gene expression and TCGA tumor microsatellite instability (MSI).

Oncogenic mutations mainly include single-gene mutations (amplifications, insertions, deletions, etc.) and translocations (fusions). We investigated the alterations of the CDK1 gene in different tumor samples from the TCGA cohort. As shown in Figure 3(a), the highest frequency of CDK1 gene alterations (>6%) was found in uterine carcinosarcomas with “amplification” as the mutation type. “Amplification” was also the main type of mutation in cholangiocarcinoma (>2%), pancreatic adenocarcinoma (<1%), stomach adenocarcinoma (<2%), breast invasive carcinoma (<2%), and esophageal adenocarcinoma (<2%). “Amplification” also occurred in lung squamous cell carcinoma (<1%), bladder urothelial carcinoma (<1%), prostate adenocarcinoma (<1%), ovarian serous cystadenocarcinoma (<1%), and head and neck squamous cell carcinoma (<1%). The “mutation” type of alteration was mainly seen in skin cutaneous melanoma (>2%) and colorectal adenocarcinoma (<2%). Kidney chromophobe, sarcoma, testicular germ cell tumors, and thyroid carcinoma were predominantly of the “deep deletion” type, with a frequency of less than 2%.  Figure 3(b) shows the types and loci of CDK1 gene alterations. We found that the “missense” mutation of CDK1 is the main type of genetic alteration. Changes in the Pkinase domain (R275Q) detected in colorectal cancer (COADREAD), BRCA, and GBM can induce a transcoding mutation of the CDK1 gene, which translates CDK1 from arginine (*R*) to glutamine (*Q*) at position 275. Subsequently, missense changes were found in CDK1 protein. The 3D structure of CDK1 showed the R275 site (Figure 3(c)).

We also analyzed the correlation between CDK1 expression and TMB and MSI in all TCGA tumors. As shown in Figure 3(d), ACC (*P*=1.01*E* − 06), BLCA (*P*=7.20*E* − 07), CHOL (*P*=0.0447), COAD (*P*=0.0025), HNSC (*P*=0.0137), KICH (*P*=0.0036), KIRC (*P*=0.0048), acute myeloid leukemia (LAML, *P*=0.0237), LGG (*P*=4.13*E* − 16), LUSC (*P*=1.15*E* − 05), PAAD (*P*=1.71*E* − 08), SARC (*P*=5.50*E* − 06), SKCM (*P*=1.31*E* − 06), UCEC (*P*=0.0099), and uterine carcinosarcoma (UCS, *P*=0.0021) in CDK1 expression were positively correlated with TMB, while CDK1 expression in thymoma (THYM, *P*=4.71*E* − 11) was negatively correlated with TMB. As shown in Figure 3(e), CDK1 expression in HNSC (*P*=0.0210), MESO (*P*=0.0307), READ (*P*=9.94*E* − 05), SARC (*P*=0.0043), STAD (*P*=1.53*E* − 13), and UCEC (*P*=2.06*E* − 10) was positively correlated with MSI, while lymphoid neoplasm diffuse large B-cell lymphoma (DLBC, *P*=0.0330) showed a negative correlation between CDK1 expression and MSI.

Pan-cancer analysis of genetic and epigenetic characteristics of CDK1 gene revealed differential mutations of CDK1 in different TCGA tumors. The correlation of CDK1 with TMB and MSI in different tumors was also analyzed to provide a basis for ICIs-based therapy.

### 3.4. The Phosphorylation of CDK1 Protein in TCGA Tumor Tissues Was Higher than That in Normal Tissues

**Figure 4:**
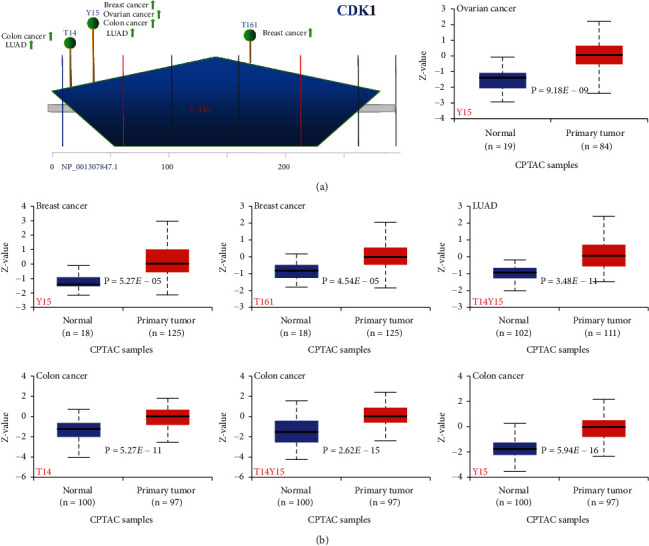
Phosphorylation analysis of CDK1 protein in different tumors. (a) Based on the CPTAC dataset, we analyzed the expression level of CDK1 phosphoprotein (NP_001307847.1, T14, Y15, and T161 sites) between normal tissue and primary tissue of selected tumors via the UALCAN. The phosphoprotein sites with positive results are displayed in the schematic diagram of the CDK1 protein. (b) Box plots of CDK1 protein phosphorylation in different tumors.

We compared the differences in CDK1 phosphorylation levels in normal and primary tumor tissues using the CPTAC dataset analysis in the UALCAN database.  Figure 4(a) summarizes the phosphorylation loci of CDK1 and the phosphorylation levels in specific cancer types. The phosphorylation loci with differences in the CDK1 S_TKc domain mainly included T14, Y15, and T161. Notably, analysis of the CDK1 amino acid sequence using the bioinformatics tool PhosphoNET (https://www.phosphonet.ca) also identified T14, Y15, and T161 as potential phosphorylation loci. Further analysis of the above loci suggested that the phosphorylation levels of the Y15 locus were higher in primary tumor tissues than in normal tissues in ovarian, breast, LUAD, and colon cancers (all *P* < 0.05) (Figure 4(b)). The phosphorylation level of the T14 locus was also significantly higher in primary tumor tissue than in normal tissue in LUAD and colon cancer. Considering that the phosphorylation levels of the Y15 locus were significantly elevated in several primary tumor tissues, further molecular testing might help to determine the potential role of Y15 phosphorylation in tumorigenesis.

Protein phosphorylation is a common process that regulates the activity of oncogenic and tumor suppressor proteins; dysregulated protein phosphorylation is often a predisposing factor for a variety of diseases. Our results showed that protein phosphorylation levels in tumor tissues were significantly higher than those in normal tissues; dysregulation of CDK1 protein phosphorylation may alter the activity of oncogenic-related signaling pathways and contribute to the formation of associated tumor phenotypes.

### 3.5. Different Methylation Levels of CDK1 in Different TCGA Tumor Tissues

**Figure 5:**
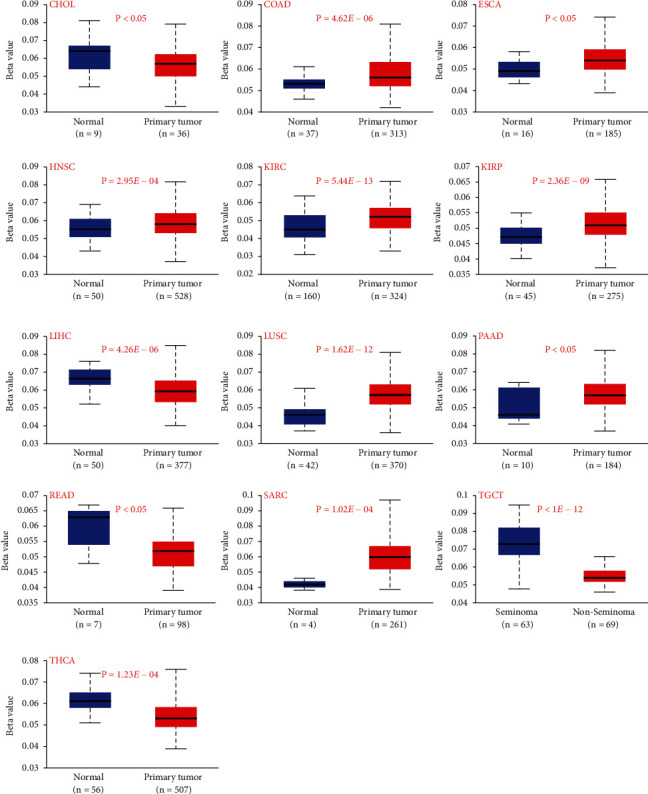
Methylation analysis of the CDK1 gene in different tumors. Box plots of methylation in different tumors, including CHOL, COAD, ESCA, HNSC, KIRC, KIRP, LIHC, LUSC, PAAD, READ, SARC, THCA, and testis germ tumor.

DNA methylation directly affects cancer development, and hence, we investigated the DNA methylation levels of CDK1 in different tumors using the TCGA dataset in the UCLAN database. As shown in Figure 5, the methylation levels of CDK1 were significantly lower in CHOL, LIHC, READ, testis germ cell tumor, and THCA tumor tissues compared to that in the normal tissues (all *P* values <0.05), whereas the methylation levels of CDK1 in COAD, ESCA, HNSC, KIRC, KIRP, LUSC, PAAD, and SARC tumor tissues were significantly higher (all *P* values <0.05). Due to the lack of CDK1 expression data, we did not analyze the relationship between DNA methylation and CDK1 expression. Related studies show that tumor suppressor genes can be suppressed by hypermethylation and oncogenes can be activated by hypomethylation, the differential expression of CDK1 phosphorylation in different TCGA tumors leads to genomic instability and accelerated tumor progression. Hypomethylation status of CDK1 in some tumors may lead to activation of other oncogenes, while hypermethylation status in other tumors may further exacerbate carcinogenesis by silencing tumor-associated suppressor genes.

### 3.6. CDK1 Expression Level Was Related to the Level of Immune Infiltration

**Figure 6:**
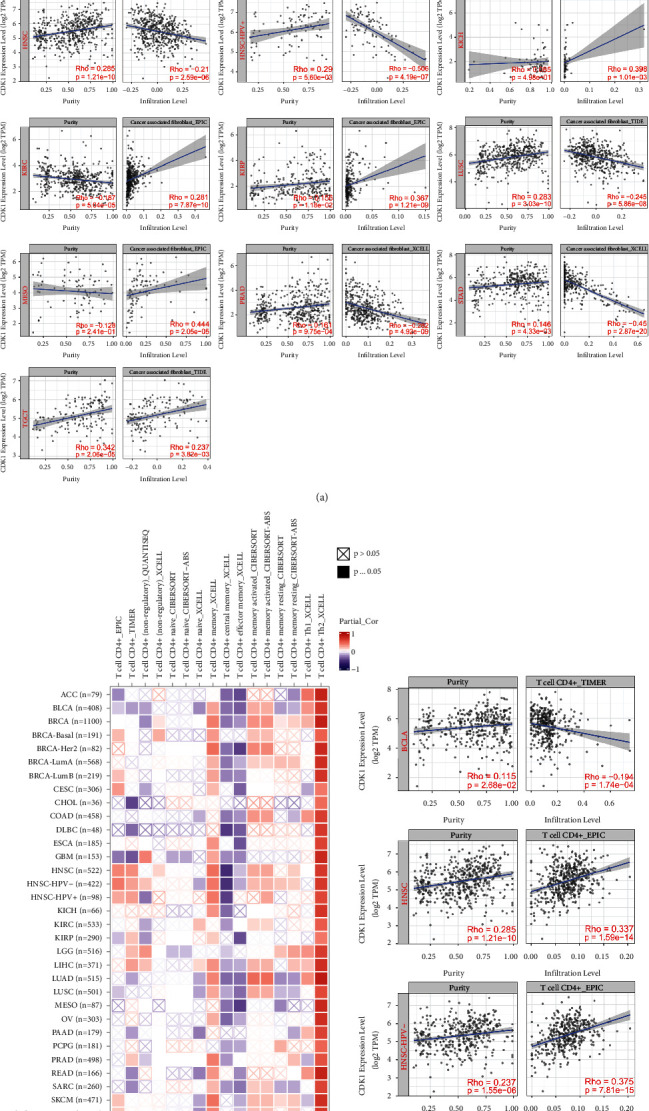
Correlation analysis between CDK1 expression and immune infiltration of tumor-associated fibroblasts and CD4+ T cells. (a) Different algorithms were used to determine the correlation between the expression level of the CDK1 gene and the infiltration level of tumor-associated fibroblasts across all types of cancer in TCGA. (b) Correlation of CDK1 expression with infiltrating levels of CD4+ T cells in different types of cancer.

Tumor-infiltrating immune cells are an important component of the tumor microenvironment, and they play an important role in tumor growth, development, and drug resistance [50]. Tumor-associated fibroblasts are one of the important cells associated with tumor malignancy and are the most prominent stromal component and key players in tumor progression [51]. We used TIDE, XCELL, MCPCOUNTER, and EPIC algorithms to investigate the relationship between the level of tumor-associated fibroblast infiltration and CDK1 gene expression in different types of TCGA tumors (Figure 6(a)). Based on all or most of the algorithms, we observed a negative correlation between CDK1 expression and the infiltration values of tumor-associated fibroblasts in COAD, BRCA-Basal, THYM, HNSC, HNSC (human papillomavirus [HPV+]), LUSC, PRAD, and STAD tumors. There was a positive correlation in KICH, KIRC, KIRP, MESO, and testicular germ cell tumor (TGCT).

We also investigated the relationship between CDK1 and other immune infiltrating cells using different algorithms such as EPIC, TIMER, QUANTISEQ, XCELL, CIBERSORT, and CIBERSORT-ABS to analyze the relationship between CDK1 expression and the infiltration levels of CD4+ T cells, CD8+ T cells, B cells, DC, and macrophages in different TCGA tumors. Specifically, as shown in Figure 6(b), CDK1 expression in BLCA and STAD was negatively correlated with the infiltration value of CD4+ T cells, whereas it was positively correlated in HNSC and HNSC-HPV F02D. Notably, in all TCGA tumors, based on the XCELL algorithm, we found a positive correlation between the infiltration values of Th2-type CD4+ T cells and the expression of CDK1. As shown in Supplementary Figure 2A, CDK1 expression in different TCGA tumors of BRCA, BRCA-lumB, HNSC, HNSC-HPV+, KIRC, LIHC, LUAD, LUSC, THCA, and THYM showed a positive correlation with the infiltration value of CD8+ T cells, while a negative correlation was observed in PAAD and UCEC. We found a positive correlation between CDK1 expression and B-cell infiltration values in HNSC, HNSC-HPV+, KIRC, LIHC, PRAD, THCA, and THYM, while CDK1 expression was negatively correlated with B-cell infiltration values in LUAD, MESO, PAAD, STAD, and TGCT (Supplementary Figure 2B). As shown in Supplementary Figure 2C, based on most algorithms, CDK1 expression in KIRC, LGG, and THYM was positively correlated with DC cell infiltration values, while it was negatively correlated in STAD and TGCT. We also observed a positive correlation between CDK1 expression and macrophage infiltration values in BLCA, BRCA, KIRC, PRAD, and THCA tumors, and a negative correlation in CESC, KIRP, LIHC, STAD, TGCT, and THYM tumors (Supplementary Figure 2D). These results provide strong evidence that the CDK1 gene might play an important role in tumor immune microenvironment, and CDK1 might be involved in the migration of immune cells to the tumor microenvironment. To analyze the correlation between CDK1 gene expression and tumor-infiltrating immune cells and to lay the foundation for the next antitumor immunotherapy based on CDK1 tumor targets.

### 3.7. The Level of CDK1 Was Positively Correlated with the Expression of RNA Methylation Regulatory Proteins in TCGA Tumors

**Figure 7:**
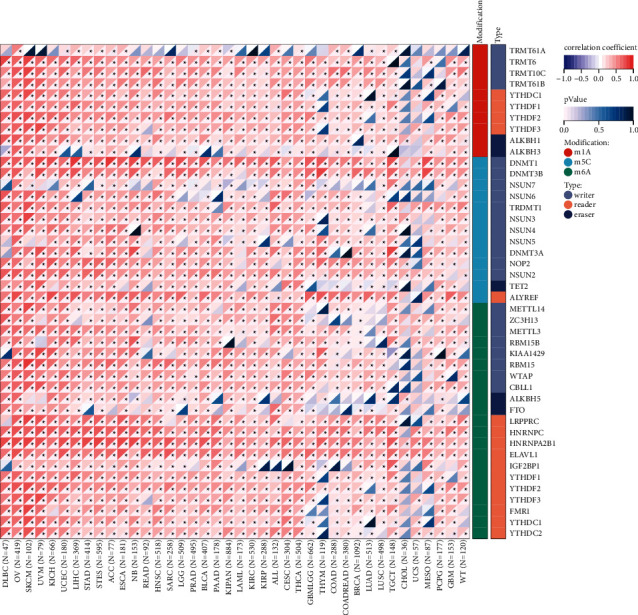
Pan-cancer correlation between CDK1 and 44 RNA methylation regulatory proteins. RNA methylation modifications are divided into three categories: m1A, m5A, and m6C; modified protein types are divided into three categories: writer, reader, and eraser.

RNA plays essential roles in not only translating nucleic acids into proteins, but also in gene regulation, environmental interactions, and many human diseases. A growing number of studies have shown that RNA methylation modification-related proteins are critical in tumor development. Our pan-cancer analysis showed (Figure 7) a significant positive correlation with expression between the CDK1 gene and three classes (*m1A*, *m5C*, *m6A*) of RNA methylation regulatory proteins. The positive pan-cancer correlations with m1A RNA methylation regulatory proteins YTHDF2 and ALKBH1, m5C methylation regulatory proteins DNMT1, DNMT3B, and ALYREF, and m6C RNA methylation regulatory proteins HNRNPC, HNRNPA2B1, and ELAVL1 were particularly significant. Our study reveals a significant positive correlation between CDK1 and RNA methylation regulatory proteins at the pan-cancer level of expression. CDK1 participates in RNA metabolic processes by affecting the expression levels of related RNA methylation regulatory proteins, this may be one of the potential mechanisms by which CDK1 exerts its corresponding oncogenic effects. Up- or downregulating the expression level of RNA methylation regulatory proteins by targeting CDK1 may become a new approach for tumor prevention and treatment.

### 3.8. Enrichment Analysis of CDK1-Related Partners

**Figure 8:**
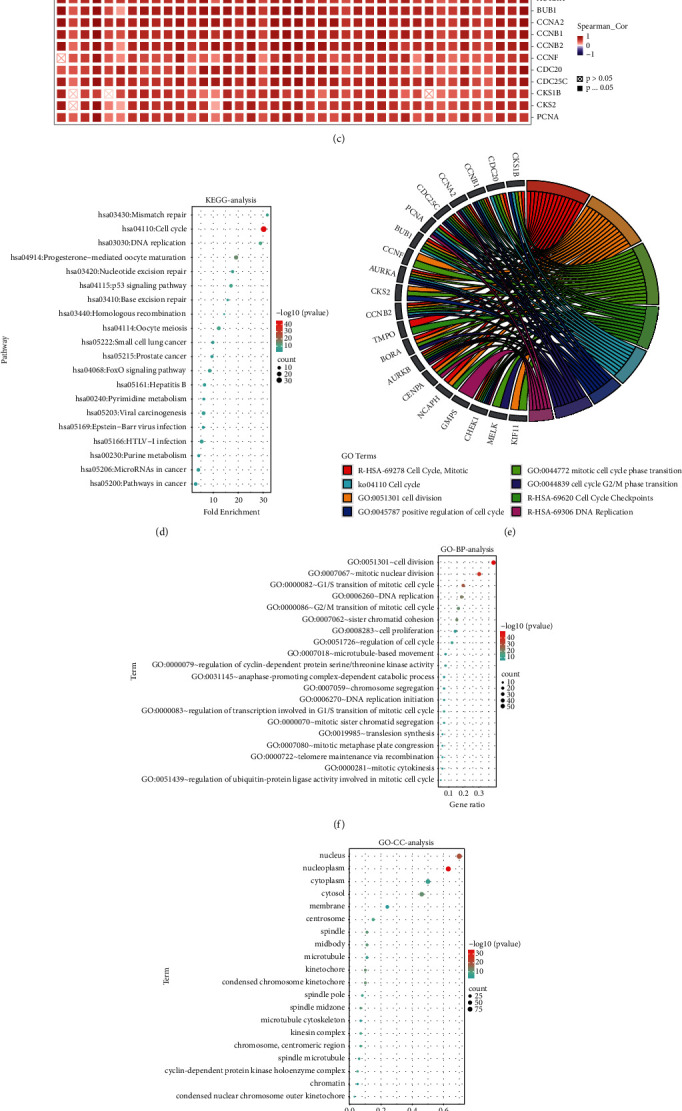
CDK1-related gene enrichment analysis. (a) PPI network analysis of CDK1-related genes. The visualizing interaction network of CDK1-binding proteins was obtained based on the STRING database. (b) Venn diagram of 50 CDK1-interacting proteins and 100 CDK1-associated genes. (c) Heat map of the gene correlation analysis. (d) KEGG analysis of CDK1-binding and interacted genes. (e) GO enrichment chord plot for the top 20 genes associated with CDK1. (f) GO-BP analysis of CDK1-binding and interacted genes. (g) GO-CC analysis of CDK1-binding and interacted genes. (h) GO-MF analysis of CDK1-binding and interacted genes.

To further investigate the mechanism of action of the CDK1 gene in tumorigenesis, we performed functional enrichment analysis of CDK1-related binding proteins and CDK1 expression-related genes. Based on the STRING tool, we obtained 50 experimentally validated CDK1-related binding proteins and constructed a protein-protein interaction (PPI) network for these proteins (Figure 8(a)). Additionally, we obtained the top 100 genes associated with CDK1 expression using the GEPIA2 database, and a cross-tabulation analysis of the two gene sets showed that 11 genes overlapped, which included CKS1B, CDC20, CCNB1, CCNA2, CDC25C, PCNA, BUB1, CCNF, AURKA, CKS2, and CCNB2 (Figure 8(b)). We also performed a pan-cancer expression correlation analysis of CDK1 and the 11 genes using the TIMER2 database and showed a positive correlation between CDK1 and the expression of these molecules in TCGA tumors (Figure 8(c)).

We combined these two gene datasets for the KEGG and GO enrichment analysis. The results of the KEGG analysis suggested that CDK1 might be involved in different pathways such as mismatch repair, cell cycle, progesterone-mediated oocyte maturation, oocyte meiosis, DNA replication, HTLV-I infection, and pathways associated with cancer (Figure 8(d)). We found that CDK1-related genes were enriched in different pathways such as cell division, mitotic nuclear division, G1/S transition of the mitotic cell cycle, DNA repair, and G2/M transition of the mitotic cell cycle in the GO enrichment analysis category of “biological processes” (Figure 8(f)). This suggested that CDK1 plays an important role in cell cycle progression. In the GO enrichment analysis category “cellular components,” CDK1-related genes were significantly enriched in different cellular components such as the nucleus, nucleoplasm, condensed chromosome kinetochore, membrane, and cytoplasm (Figure 8(g)). In the GO enrichment analysis category of “molecular function,” the role of CDK1 in tumor pathogenesis might be related to protein binding, protein kinase binding, and ATP binding (Figure 8(h)). We have shown the relevant functional pathways involved in the top 20 genes associated with CDK1 as a chord plot (Figure 8(e)). The corresponding enrichment analysis of CDK1 further revealed the molecular mechanisms involved in its oncogenic role, which is involved in the malignant progression of tumors by affecting related signaling pathways or cellular functions.

### 3.9. In Vitro Experiments Verify the High Expression of CDK1 in Tumor Tissues

**Figure 9:**
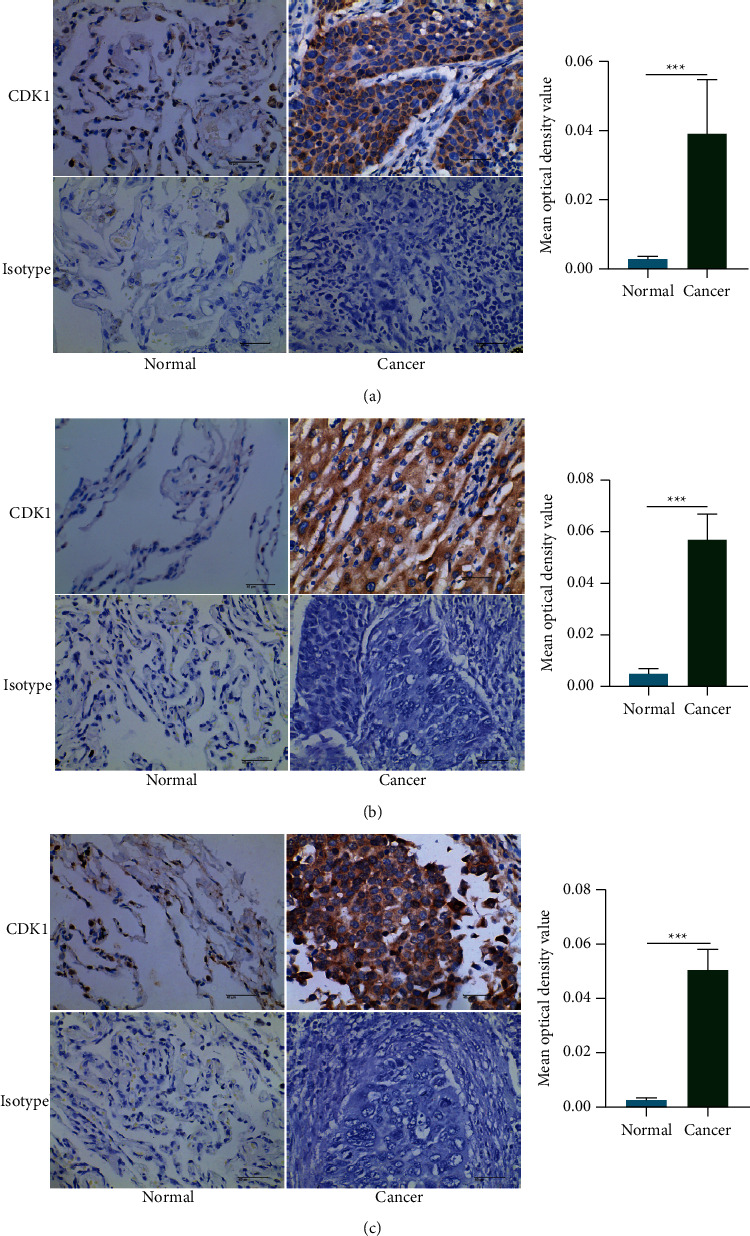
IHC verification of CDK1 gene expression in tumor tissues and normal tissues. (a) Expression of CDK1 gene in lung cancer tissues and normal tissues. (b) Expression of CDK1 gene in hepatocellular carcinoma tissues and normal tissues. (c) Expression of CDK1 gene in breast cancer tissues and normal tissues (*n* = 10 per CDK1 experiment group, *n* = 5 per isotype group; ns, not statistically significant; ^*∗*^*P* < 0.05; ^*∗∗*^*P* < 0.01; and ^*∗∗∗*^*P* < 0.001. The Student's *t* test was used for statistical analysis).

To further elucidate the difference of CDK1 gene expression in tumor tissues and normal tissues, we collected human lung cancer, liver cancer, and breast cancer tissues along with the adjacent normal tissues from the Affiliated Hospital of Southwest Medical University and conducted experimental studies on human lung cancer, liver cancer, and breast cancer tissue samples using immunohistochemical methods. The Image-pro Plus6.0 software was used for the semiquantitative analysis of different tissue specimens. Using the area of the whole image for measurement, the mean optical density values of CDK1-positive expression in the corresponding cancer tissues of lung cancer, liver cancer, and breast cancer, as well as the normal tissues, were calculated. The results showed that CDK1 was highly expressed in lung cancer tissues (Figure 9(a)), liver cancer tissues (Figure 9(b)), and breast cancer tissues (Figure 9(c)), and the difference in expression between the cancer tissues and normal tissues was significant. In contrast, there was no obvious positive staining in the rabbit IgG isotype control group. In vitro experiments confirmed the high expression status of CDK1 in tumor tissues, adding credibility to its use as a potential therapeutic target for differentially highly expressed tumors.

## 4. Discussion

As an important member of the cyclin-dependent kinase family, CDK1 plays a critical role in cell cycle regulation, immune checkpoint activation, and DNA damage repair. As an important locus of signaling pathways, the CDK1 gene is essential for tumor initiation and progression in different types of cancer, promoting the progression of malignancy through different signaling pathways [52, 53]. The CDK1 protein structure is conserved from yeast to humans, suggesting that similar mechanisms may exist for the normal physiological roles of CDK1. Whether CDK1 plays a role in the pathogenesis of different tumors through some common molecular mechanisms is unknown. There are no reports of pan-cancer analysis of CDK1 from an overall perspective. Therefore, using data from various databases such as TCGA, GEO, and CPTAC, we revealed the molecular characteristics of the CDK1 gene at multiple levels, including gene expression, gene alteration, DNA methylation, and protein phosphorylation. We conducted a comprehensive investigation on the bioinformatics of the CDK1 gene in 33 tumors to elucidate its functions in the development of different tumors and potential regulatory pathways.

In this study, we first investigated the expression of CDK1 in the pan-cancer dataset (Figures 1(a)–1(d)), Supplementary Table 1). The results of the analysis of different datasets showed that the CDK1 gene was highly expressed in most tumors. CDK1 expression in different tumors was analyzed using the Oncomine database, the TIMER database, and the combined TCGA and GTEx datasets to avoid bias in the results of single dataset analysis. We also conducted in vitro experiments to select human lung, liver, and breast cancer tissues for immunohistochemical semiquantitative analysis to further validate our findings (Figure 9). Tumorigenesis is usually accompanied by abnormal gene expression, and this altered expression contributes to the development of tumors [54]. CDK1 expression and subcellular localization are regulated by RAR*γ* and its expression level is usually positively correlated with the activation of Wnt/*β*-catenin [55–57]. We also found that high CDK1 expression was associated with the clinicopathological staging of BRCA, COAD, LUAD, and LUSC (Figure 1(e)).

In this study, we used independent datasets from TCGA, Kaplan-Meier plotter, and PrognoScan to determine the relationship between CDK1 expression levels and pan-cancer prognosis (Figure 2, Supplementary Figure 1). In different datasets, based on all or most tumor types, high CDK1 expression levels suggested a poor prognosis for tumor patients, affecting OS, DFS, DMFS, RFS, DRFS, DSS, and MFS survival progression in tumor patients. Previous studies have shown that high CDK1 expression is significantly associated with reduced overall survival in patients with colon cancer [58] and hepatocellular carcinoma [59]. These results are consistent with our current findings. Notably, analysis from the Kaplan-Meier plotter dataset showed that high CDK1 expression was significantly associated with improved OS in blood cancers and improved DFS in colorectal cancers. The results of PrognoScan analysis suggested that patients with CDK1-positive esophageal squamous cell carcinoma had better OS and RFS. There were no reports on the effect of CDK1 on survival progression in blood cancer, colorectal cancer, and esophageal squamous cell carcinoma. In our study, high expression of CDK1, as a protective factor, was found to prolong the survival of patients with these tumors. However, this observation needs to be confirmed with larger sample size and through other clinical characteristics of the tumor patients. Taken together, these findings provide insights into the application of CDK1 as a prognostic marker for pan-cancer in the context of immuno-oncology, thus contributing to the potential development of research on CDK1 gene-targeted therapy.

TMB refers to the number of nonsynonymous mutations in somatic cells within a given genome, which can indirectly reflect the ability and extent of neoantigen production by tumors. TMB is a potential biomarker of response to ICIs and can predict the efficacy of immunotherapy for a variety of tumors [60]. Clinical studies have shown that high TMB is associated with improved responses and survival benefits in cancer patients after ICI treatment [61–63]. In tumors, MSI is a relatively common phenomenon. The status of MSI predicts the cause and development of tumors and also plays an important role in different cancer types as an aid to diagnosis and drug guidance. A comprehensive MSI screening study showed that the degree of MSI was positively correlated with survival in cancer patients and that MSI-positive tumors generally had a better prognosis than MSI-negative tumors [64, 65]. We found that CDK1 expression was positively correlated with TMB in 15 cancers and with MSI in five cancers (Figure 3(d)). Therefore, we hypothesized that tumors with high CDK1 expression and positive TMB and MSI might have a greater survival benefit after treatment with immune checkpoint inhibitors (Figure 3(e)).

The survival, growth, migration, and dormancy of tumor cells are influenced by the surrounding tumor microenvironment, which is important for tumor progression [66–68]. In the tumor microenvironment, tumor-associated fibroblasts are a major component of the tumor stroma and are currently considered to be one of the most active cell types in the tumor microenvironment, playing a central role in tumorigenesis, progression, and metastasis [69–71]. Tumor-associated fibroblasts accumulated in the tumor microenvironment might promote the growth and migration of a variety of solid malignancies, including breast cancer [72], esophageal cancer [73], bladder cancer [74], gallbladder cancer [75], and bile duct cancer [76]. We found that CDK1 expression was negatively correlated with infiltration values of tumor-associated fibroblasts in COAD, BRCA-Basal, THYM, HNSC, HNSC-HPV+, LUSC, PRAD, and STAD tumors, whereas it was positively correlated in KICH, KIRC, KIRP, MESO, and TGCT. Meanwhile, we demonstrated the high expression status of the CDK1 gene in COAD, THYM, HNSC, HNSC-HPV+, LUSC, PRAD, STAD, KICH, KIRC, KIRP, MESO, and TGCT tumors (Figure 6(a)). Whether the CDK1 gene and tumor-associated fibroblasts have competitive inhibitory or synergistic effects on promoting tumor cell proliferation and the progression of malignancy in these tumors is unclear, and the mechanism of action between the two needs a detailed investigation.

Interestingly, we found a positive correlation between the infiltration values of Th2-type CD4+ T cells and CDK1 expression in all TCGA tumors (Figure 6(b)). There is growing evidence that CD4+ T cells play a central role in the initiation and maintenance of the immune response against cancer or autoimmune diseases [77, 78]. As an essential component of the tumor microenvironment, it exerts powerful antitumor effects by recognizing tumor-associated MHC class II molecules. However, most solid tumors do not express MHC class II molecules, which limit the ability of CD4+ T cells to act at the tumor site [79]. CD4+ T cells are mainly composed of different cell subsets such as Th1, Th2, Th17, and Treg. Among them, Th1 cells assist CD8+ T cells in mediating immunity against tumors and viruses [80]. Th2 cells are best known for enhancing immunity against parasites, and their pathogenic role in allergic diseases has been well documented [81, 82]. However, the role of Th2 cells in the antitumor immune response is not well-understood. Th2 cells mainly secrete IL-4, IL-5, IL-6, and IL-10 cytokines [83]. IL-10 cytokines produced by Th2 cells directly inhibit Th1 cells and indirectly inhibit the activity of Th2 cells [84, 85]. Whether the high expression of CDK1 breaks the Th1/Th2 balance and increases Th2-type CD4+ T cells, leading to immune dysfunction and the suppression of the antitumor effects of Th1, needs further investigation. Our study did not find a significant effect of CDK1 expression on tumor infiltration in Th1-type CD4+ T cells. Additionally, we found that CDK1 gene expression in different tumors correlated with the infiltration of CD8+ T cells, B cells, DC cells, and macrophages. This association between CDK1 and the tumor microenvironment might be another reason for the prognostic significance of CDK1 in various cancers, where the aberrant expression of CDK1 could play a dominant role in the tumor microenvironment.

Some studies have reported that targeting CDK1 can improve the effectiveness of antitumor immunotherapy or reverse chemotherapy resistance and prolong survival time. The results of Jin Huang et al. showed that CDK1 kinase activity plays an important role in IFNG-mediated tumor immune escape. Inhibition of the kinase activity of CDK1 can prevents the expression of relevant immune checkpoints, alters the tumor microenvironment, and can significantly improve the overall survival rate in a mouse pancreatic cancer tumor model [86]. A recent report also showed that ATR inhibits CDK1-SPOP signaling and thus enhances anti-PD-L1 cytotoxicity in prostate cancer. Combination of ATR inhibitor and anti-PD-L1 therapy produces potent innate immune activation and a synergistic T-cell-dependent therapeutic response [87]. Meanwhile, CDK1 plays an important role in reversing chemotherapy resistance and improving the effectiveness of chemotherapeutic drugs. The application of CDK1 inhibitors has been reported to improve the efficacy of the chemotherapeutic drug sorafenib targeting tumor stem cells in the treatment of hepatocellular carcinoma, anti-CDK1 combination chemotherapy significantly inhibits tumor growth in hepatocellular carcinoma, while being able to overcome resistance to sorafenib [59]. Also, it has been shown that the use of CDK1 inhibitors can interfere with the proliferation of gastrointestinal mesenchymal tumor cells with high CDK1 expression. More importantly, anti-CDK1 inhibitor treatment reduced tumor growth in imatinib-resistant and imatinib-sensitive gastrointestinal mesenchymal tumor xenograft mice models, reversing the chemoresistant and sensitive situation [15]. Based on the importance of CDK1 in antitumor immunotherapy and reversal of chemotherapy resistance, CDK1 can be an important factor in measuring the efficacy of tumor immunotherapy and chemotherapy.

We investigated the function of differentially expressed CDK1 by GO enrichment analysis and KEGG pathway enrichment analysis (Figures 8(d)–8(h)). We found that differentially expressed CDK1 is mainly associated with the regulation of the cell cycle, mismatch repair, DNA replication, G1/S and G2/M transitions of the mitotic cell cycle, protein binding, protein kinase binding, and ATP binding. Previously, CDK1 was shown to play a key role in cell cycle progression [85], which was consistent with our findings. By analyzing CDK1-related genes, we found that the positive association of CKS1B, CDC20, CCNB1, CCNA2, CDC25C, PCNA, BUB1, CCNF, AURKA, CKS2, and CCNB2 with the CDK1 gene was consistent in all TCGA tumors (Figure 8(c)). This suggested that CDK1-related enrichment pathways could serve as the underlying markers to identify patients in need of therapy.

In summary, our pan-cancer analysis of CDK1 showed a significant correlation between CDK1 expression and clinical prognosis, DNA methylation, protein phosphorylation, immune cell infiltration, RNA methylation regulatory proteins, tumor mutational load, and microsatellite instability in multiple tumors. In this study, we determined the role of CDK1 in tumorigenesis from the perspective of clinical tumor samples. Based on our established findings, the expression pattern as well as the functional importance of CDK1 make it a promising target for clinical antitumor therapy. The inclusion of CDK1 in tumor marker testing is of great significance, as well as the development of new anti-CDK1 drugs targeting CDK1, anti-CDK1 combined with immunotherapy or combined with chemotherapy, making it possible to extend the median survival time of tumor patients.

## Data Availability

The original contributions presented in the study are included in the article/supplementary material. Further inquiries can be directed to the corresponding author/s.

## Abbreviations

CDK1: Cyclin-dependent kinase 1
TCGA: The Cancer Genome Atlas
GEO: Gene Expression Omnibus
OS: Overall survival
DFS: Disease-free survival
DMFS: Distant metastasis-free survival
RFS: Recurrence-free survival
DRFS: Distant recurrence-free survival
DSS: Disease-specific survival
TMB: Tumor mutational burden
MSI: Microsatellite instability
CDKs: Cyclin-dependent kinases
CDC2: Cell division cycle 2
GTEx: Genotype-tissue expression
CPTAC: Clinical Proteomic Tumor Analysis Consortium
RCC: Renal clear cell carcinoma
UCEC: Uterine corpus endometrial carcinoma
LUAD: Lung adenocarcinoma
CNA: Copy number alteration
GDC: Genomic data commons
DC: Dendritic cells
COR: Correlation coefficients
KEGG: Kyoto Encyclopedia of Genes and Genomes
GO: Gene ontology
BP: Biological process
CC: Cellular component
MF: Molecular function
BLCA: Bladder urothelial carcinoma
BRCA: Breast invasive carcinoma
CESC: Cervical squamous cell carcinoma and endocervical adenocarcinoma
CHOL: Cholangiocarcinoma
COAD: Colon adenocarcinoma
ESCA: Esophageal carcinoma
GBM: Glioblastoma multiforme
HNSC: Head and neck squamous cell carcinoma
KIRC: Kidney renal clear cell carcinoma
LIHC: Liver hepatocellular carcinoma
LUSC: Lung squamous cell carcinoma
PCPG: Pheochromocytoma and paraganglioma
PRAD: Prostate adenocarcinoma
READ: Rectum adenocarcinoma
STAD: Stomach adenocarcinoma
THCA: Thyroid carcinoma
KICH: Kidney chromophobe
ACC: Adrenocortical carcinoma
KIRP: Kidney renal papillary cell carcinoma
LGG: Brain lower grade glioma
MESO: Mesothelioma
PAAD: Pancreatic adenocarcinoma
SARC: Sarcoma
SKCM: Skin cutaneous melanoma
UVM: Uveal melanoma
COADREAD: Colorectal cancer
LAML: Acute myeloid leukemia
UCS: Uterine carcinosarcoma
THYM: Thymoma
DLBC: Lymphoid neoplasm diffuse large B-cell lymphoma
HPV: Human papillomavirus
TGCT: Testicular germ cell tumor
PPI: Protein-protein interaction
m1A: 1‐Methyladenosine
m5C: 5‐Methylcytidine
m6A: 6‐Methyladenosine.

## Additional Points

The “guarantor statement” does not apply to this manuscript.

## Supplementary Materials

Supplementary Materials Supplementary Table 1. Expression of CDK1 in different types of lung, stomach, liver, colon, breast, esophageal, and pancreatic cancer tissues and normal tissues. Supplementary Figure 1. Kaplan-Meier survival curves comparing the high and low expression of CDK1 in different types of cancer in the (A) PrognoScan database and (B) Kaplan-Meier plotter database. Supplementary Figure 2. Correlation between CDK1 expression in different types of cancer and infiltrating levels of (A) CD8+ T cells, (B) B cells, (C) dendritic cells, and (D) macrophages.
