# Supplementary material for: Systematic Pan-Cancer Analysis Identifies CDK1 as an Immunological and Prognostic Biomarker
Source: J Oncol. 2022 Aug 31;2022:8115474. doi: 10.1155/2022/8115474 (PMC9452984; doi:10.1155/2022/8115474)

**Supplementary Materials**

**Supplementary Table. 1** Expression of CDK1 in different types of lung, stomach, liver, colon, breast, esophageal, and pancreatic cancer tissues and normal tissues.

| **The mRNA levels of CDK1 in different types of lung cancer tissues and normal tissues at transcriptome level** | | | | | |
| --- | --- | --- | --- | --- | --- |
|  |  | **Fold** |  |  |  |
| **Name** | **Types of lung cancer VS. Nomal tissues** | **Change** | **P Value** | **t-test** | **Reference or Source** |
| **CDK1** | Squamous Cell Lung Carcinoma | 2.416 | 4.61E-07 | 13.742 | Wachi S^[1]^ |
|  | Small Cell Lung Carcinoma | 2.14 | 2.71E-08 | 6.445 | Bhattacharjee A^[2]^ |
|  | Squamous Cell Lung Carcinoma | 19.638 | 1.43E-08 | 7.164 | Bhattacharjee A^[2]^ |
|  | Small Cell Lung Carcinoma | 10.178 | 6.24E-07 | 7.243 | Bhattacharjee A^[2]^ |
|  | Lung Adenocarcinoma | 3.795 | 3.25E-04 | 4.077 | Bhattacharjee A^[2]^ |
|  | Squamous Cell Lung Carcinoma | 4.957 | 4.49E-06 | 5.577 | Bhattacharjee A^[2]^ |
|  | Large Cell Lung Carcinoma | 2.988 | 1.34E-05 | 6.04 | Bittner M |
|  | Squamous Cell Lung Carcinoma | 6.839 | 4.26E-24 | 18.338 | Hou J^[3]^ |
|  | Lung Adenocarcinoma | 3.301 | 3.81E-15 | 9.859 | Hou J^[3]^ |
|  | Large Cell Lung Carcinoma | 6.059 | 1.34E-08 | 8.615 | Hou J^[3]^ |
|  | Squamous Cell Lung Carcinoma | 5.905 | 4.85E-07 | 12.634 | Garber ME^[4]^ |
|  | Large Cell Lung Carcinoma | 5.921 | 2.05E-04 | 7.769 | Garber ME^[4]^ |
|  | Lung Adenocarcinoma | 3.215 | 2.51E-06 | 7.293 | Garber ME^[4]^ |
|  | Small Cell Lung Carcinoma | 5.686 | 0.004 | 4.939 | Garber ME^[4]^ |
|  | Squamous Cell Lung Carcinoma | 3.165 | 2.24E-07 | 5.855 | Kuner R^[5]^ |
|  | Lung Adenocarcinoma | 3.126 | 4.62E-09 | 7.118 | Su LJ^[6]^ |
|  | Lung Adenocarcinoma | 2.605 | 1.65E-17 | 10.873 | Landi MT^[7]^ |
|  | Squamous Cell Lung Carcinoma | 2.065 | 1.48E-07 | 6.1 | TCGA |
|  |  |  |  |  |  |
| **The mRNA levels of CDK1 in different types of gastric cancer tissues and normal tissues at transcriptome level** | | | | | |
|  |  | **Fold** |  |  |  |
| **Name** | **Types of gastric cancer VS. Nomal tissues** | **Change** | **P Value** | **t-test** | **Reference or Source** |
| **CDK1** | Gastric Intestinal Type Adenocarcinoma | 2.096 | 2.48E-06 | 4.799 | Ooi CH^[8]^ |
|  | Gastric Cancer | 2.526 | 1.57E-08 | 5.824 | Cui J^[9]^ |
|  | Gastric Intestinal Type Adenocarcinoma | 2.544 | 7.42E-13 | 9.474 | Chen X^[10]^ |
|  | Gastric Intestinal Type Adenocarcinoma | 3.754 | 7.51E-12 | 8.585 | D’Errico M^[11]^ |
|  | Diffuse Gastric Adenocarcinoma | 2.922 | 0.002 | 3.683 | D’Errico M^[11]^ |
|  | Gastric Mixed Adenocarcinoma | 3.951 | 7.69E-04 | 5.023 | D’Errico M^[11]^ |
|  | Gastric Intestinal Type Adenocarcinoma | 3.728 | 2.12E-05 | 4.868 | Forster S^[12]^ |
|  | Gastric Mixed Adenocarcinoma | 2.117 | 1.60E-04 | 4.89 | Cho JY^[13]^ |
|  |  |  |  |  |  |
| **The mRNA levels of CDK1 in different types of liver cancer tissues and normal tissues at transcriptome level** | | | | | |
|  |  | **Fold** |  |  |  |
| **Name** | **Types of liver cancer VS. Nomal tissues** | **Change** | **P Value** | **t-test** | **Reference or Source** |
| **CDK1** | Hepatocellular Carcinoma | 4.148 | 6.41E-29 | 13.87 | Chen X^[14]^ |
|  | Hepatocellular Carcinoma | 2.408 | 0.007 | 3.354 | Chen X^[14]^ |
|  | Hepatocellular Carcinoma | 5.573 | 1.05E-84 | 28.109 | Roessler S^[15]^ |
|  | Hepatocellular Carcinoma | 8.68 | 5.74E-10 | 7.891 | Wurmbach E^[16]^ |
|  | Hepatocellular Carcinoma | 6.782 | 1.26E-11 | 9.07 | Wurmbach E^[16]^ |
|  | Hepatocellular Carcinoma | 5.808 | 9.36E-10 | 9.077 | Roessler S^[15]^ |
|  |  |  |  |  |  |
| **The mRNA levels of CDK1 in different types of colorectal cancer tissues and normal tissues at transcriptome level** | | | | | |
|  | **Types of colorectal cancer VS. Nomal** | **Fold** |  |  |  |
| **Name** | **tissues** | **Change** | **P Value** | **t-test** | **Reference or Source** |
| **CDK1** | Rectal Mucinous Adenocarcinoma | 2.659 | 1.94E-06 | 7.501 | TCGA |
|  | Cecum Adenocarcinoma | 2.183 | 2.71E-11 | 8.781 | TCGA |
|  | Colon Mucinous Adenocarcinoma | 2.695 | 7.81E-10 | 7.695 | TCGA |
|  | Rectosigmoid Adenocarcinoma | 4.507 | 2.54E-04 | 9.681 | TCGA |
|  | Rectal Adenocarcinoma | 2.897 | 1.96E-12 | 11.1 | TCGA |
|  | Colon Adenocarcinoma | 2.274 | 6.34E-13 | 10.804 | TCGA |

| **The mRNA levels of CDK1 in different types of breast cancer tissues and normal tissues at transcriptome level** | | | | | |
| --- | --- | --- | --- | --- | --- |
|  |  | **Fold** |  |  |  |
| **Name** | **Types of breast cancer VS. Nomal tissues** | **Change** | **P Value** | **t-test** | **Reference or Source** |
| **CDK1** | Invasive Ductal Breast Carcinoma | 3.275 | 4.98E-135 | 45.054 | Curtis C^[17]^ |
|  | Invasive Lobular Breast Carcinoma | 2.325 | 2.36E-48 | 18.721 | Curtis C^[17]^ |
|  | Invasivel Breast Carcinoma | 3.396 | 1.90E-08 | 8.405 | Curtis C^[17]^ |
|  | Medullary Breast Carcinoma | 4.03 | 3.78E-15 | 13.152 | Curtis C^[17]^ |
|  | Invasive Ductal and Invasive Lobular Breast |  |  |  |  |
|  | Carcinoma | 2.931 | 1.42E-31 | 16.31 | Curtis C^[17]^ |
|  | Mucinous Breast Carcinoma | 2.476 | 7.32E-18 | 13.474 | Curtis C^[17]^ |
|  | Breast Carcinoma | 2.958 | 3.55E-06 | 7.036 | Curtis C^[17]^ |
|  | Tubular Breast Carcinoma | 2.523 | 1.03E-23 | 13.953 | Curtis C^[17]^ |
|  | Ductual Breast Carcinoma in Situ | 2.681 | 2.74E-04 | 5.16 | Curtis C^[17]^ |
|  | Invasivel Breast Carcinoma | 5.176 | 6.31E-35 | 16.809 | TCGA |
|  | Invasive Ductal Breast Carcinoma | 5.857 | 1.51E-47 | 25.541 | TCGA |
|  | Invasive Lobular Breast Carcinoma | 4.761 | 8.14E-17 | 10.614 | TCGA |
|  | Intraductal Cribriform Breasr |  |  |  |  |
|  | Adenocarcinoma | 2.05 | 8.25E-09 | 8.461 | TCGA |
|  | Mucinous Breast Carcinoma | 8.868 | 8.74E-05 | 10.841 | TCGA |
|  | Male Breast Carcinoma | 6.998 | 1.09E-05 | 13.62 | TCGA |
|  | Ductual Breast Carcinoma | 27.014 | 1.05E-09 | 13.519 | Richardson AL^[18]^ |
|  | Invasivel Breast Carcinoma | 2.123 | 5.67E-08 | 16.331 | Gluck S^[19]^ |
|  | Ductal Breast Carcinoma in Situ Epithelia | 8.92 | 2.06E-05 | 5.234 | Ma XJ^[20]^ |
|  | Invasive Ductal Breast Carcinoma Epithelia | 8.326 | 5.29E-05 | 4.764 | Ma XJ^[20]^ |
|  | Ductal Breast Carcinoma in Situ Stroma | 2.529 | 0.005 | 2.818 | Ma XJ^[20]^ |
|  | Ductual Breast Carcinoma | 4.05 | 0.008 | 4.49 | Sorlie T^[21]^ |

| **The mRNA levels of CDK1 in different types of esophageal cancer tissues and normal tissues at transcriptome level** | | | | | |
| --- | --- | --- | --- | --- | --- |
|  |  | **Fold** |  |  |  |
| **Name** | **Types of esophageal cancer VS. Nomal tissues** | **Change** | **P Value** | **t-test** | **Reference or Source** |
| **CDK1** | Esophageal Squamous Cell Carcinoma | 2.929 | 1.54E-26 | 14.271 | Su H^[22]^ |
|  | Esophageal Squamous Cell Carcinoma | 3.76 | 3.52E-11 | 10.397 | Hu N^[23]^ |

| **The mRNA levels of CDK1 in different types of pancreatic cancer tissues and normal tissues at transcriptome level** | | | | | |
| --- | --- | --- | --- | --- | --- |
|  | **Types of pancreatic cancer VS. Nomal** | **Fold** |  |  |  |
| **Name** | **tissues** | **Change** | **P Value** | **t-test** | **Reference or Source** |
| **CDK1** | Pancreatic Adenocarcinoma |  |  |  | Lacobuzio-Donahue |
|  |  | 7.707 | 3.75E-07 | 9.162 | CA^[24]^ |
|  | Pancreatic Ductal Adenocarcinoma |  |  |  |  |
|  | Epithelia | 4.942 | 0.006 | 2.805 | Grutzmann R^[25]^ |
|  | Pancreatic Carcinoma | 3.888 | 1.37E-07 | 6.564 | Pei H^[26]^ |

**Supplementary Figure 1.** Kaplan-Meier survival curves comparing the high and low expression of CDK1 in different types of cancer in the **(A)** PrognoScan database and **(B)** Kaplan-Meier plotter database.


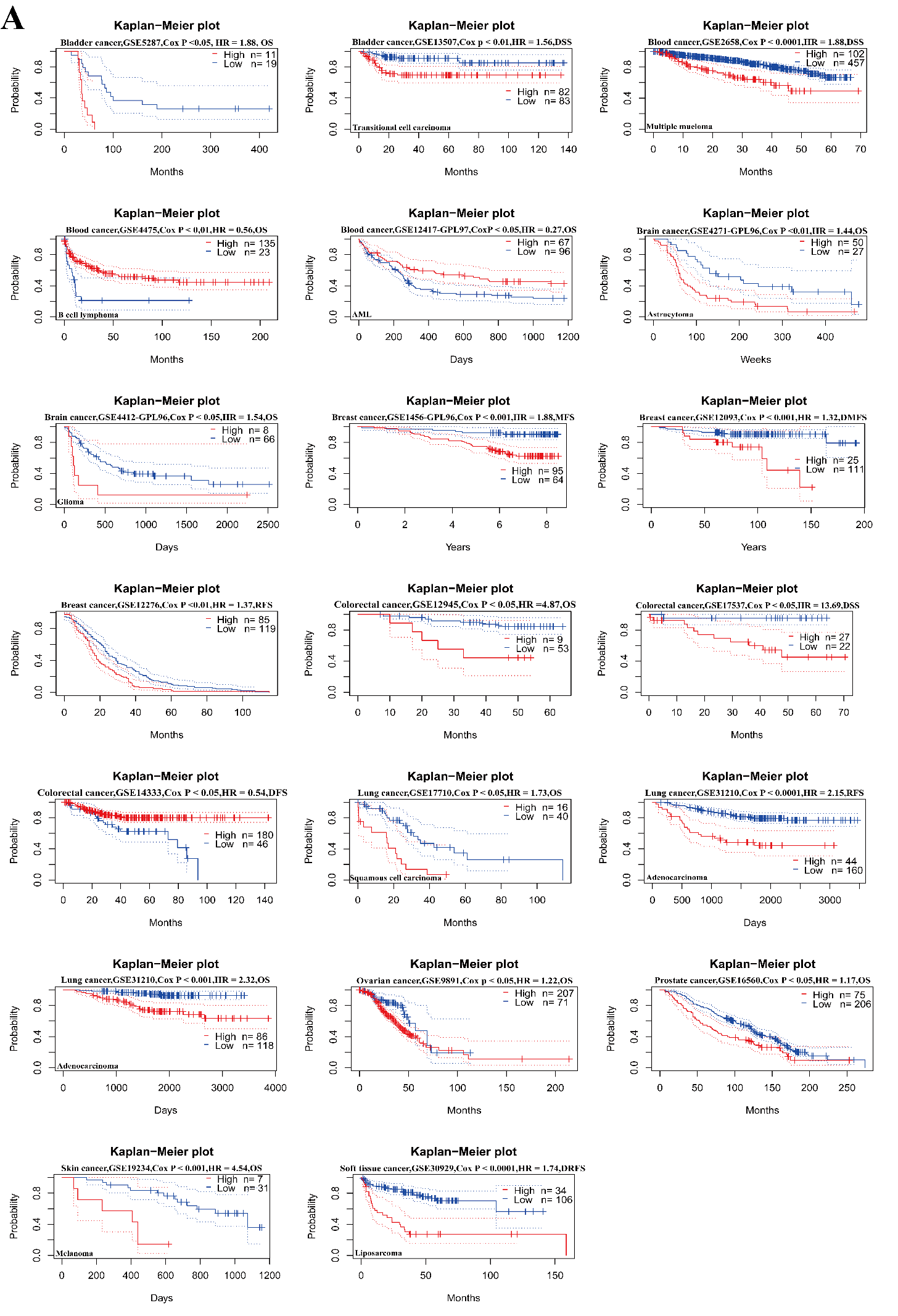


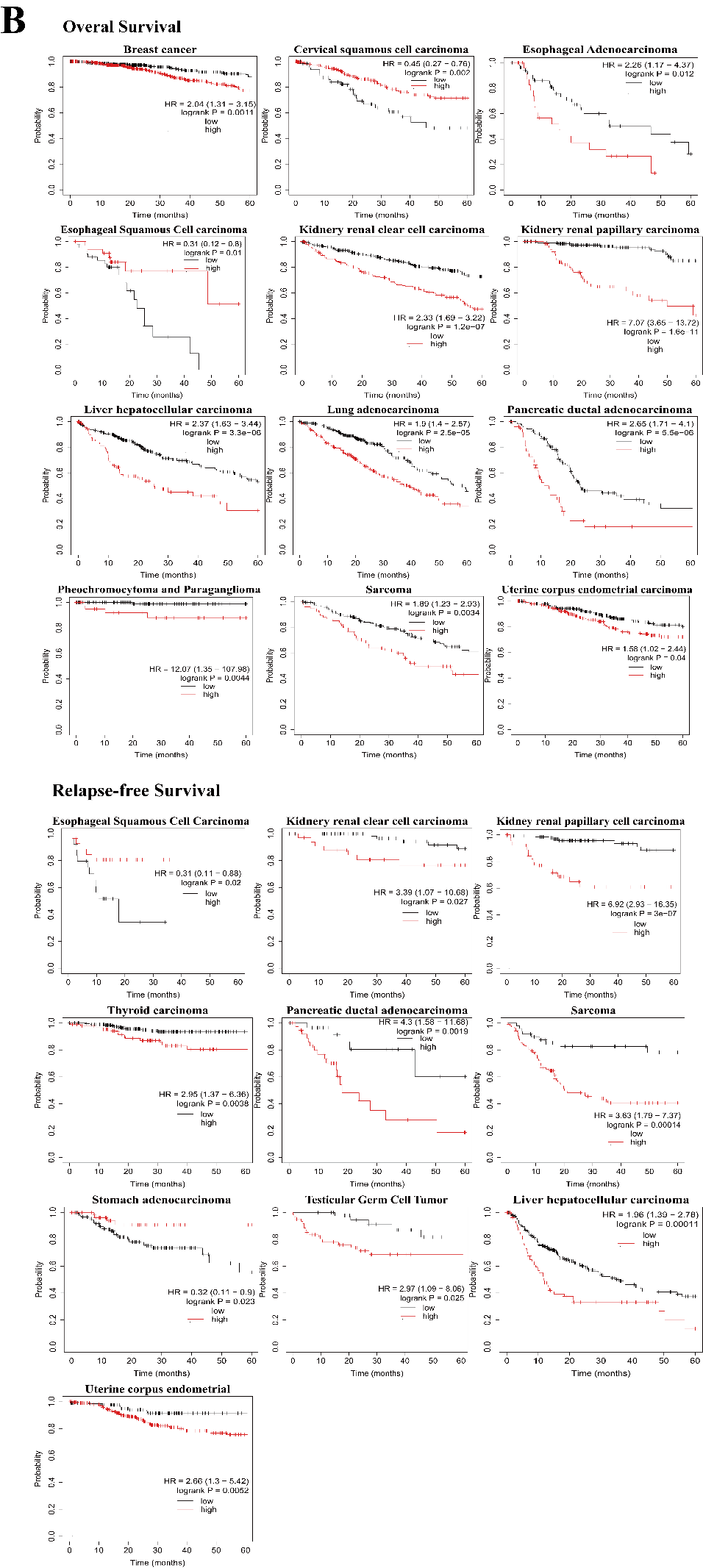


**Supplementary Figure 2.** “Correlation between CDK1 expression in different types of cancer and infiltrating levels of **(A)** CD8+ T cells, **(B)** B cells, **(C)** dendritic cells, and **(D)** macrophages.”


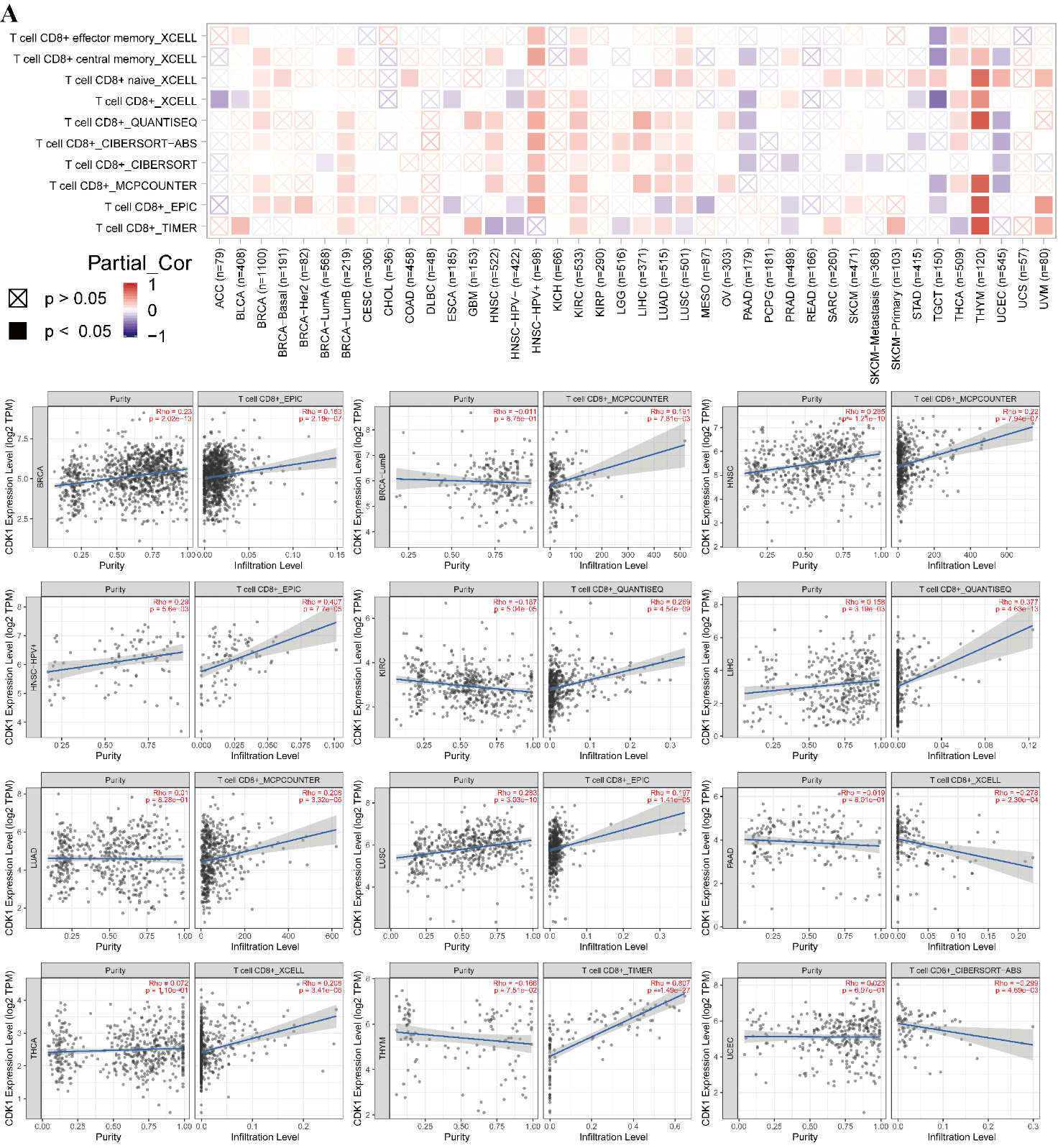


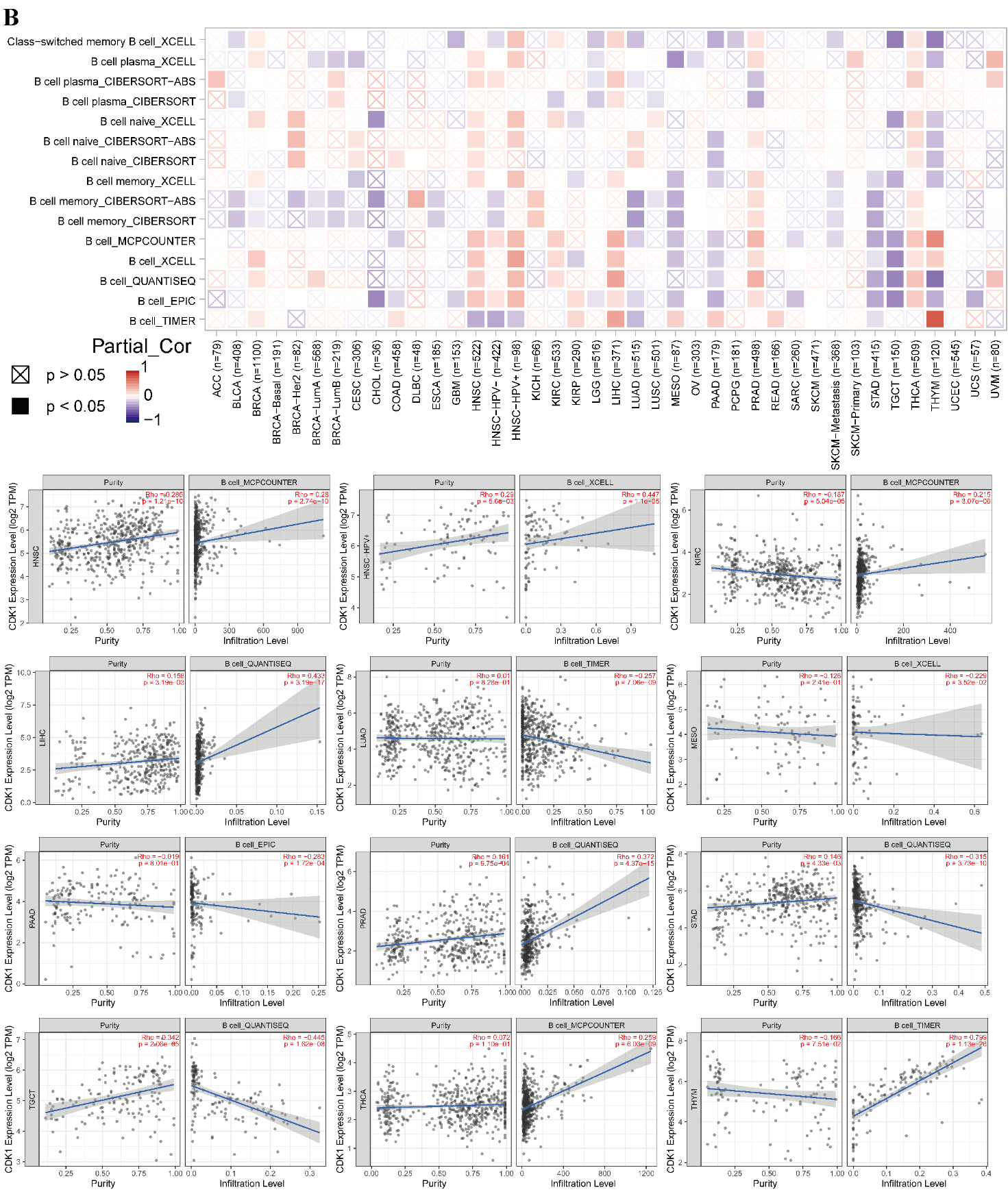


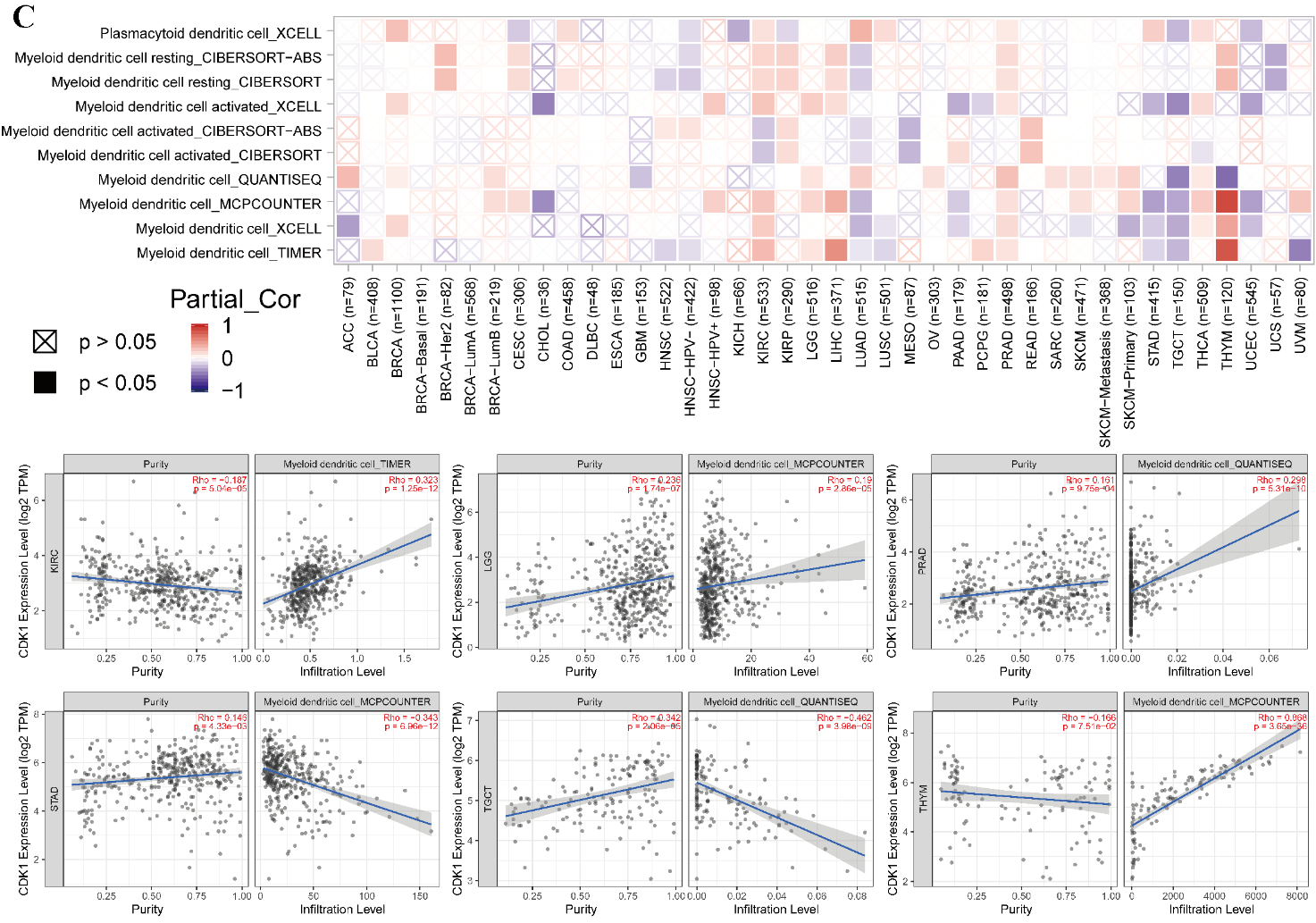


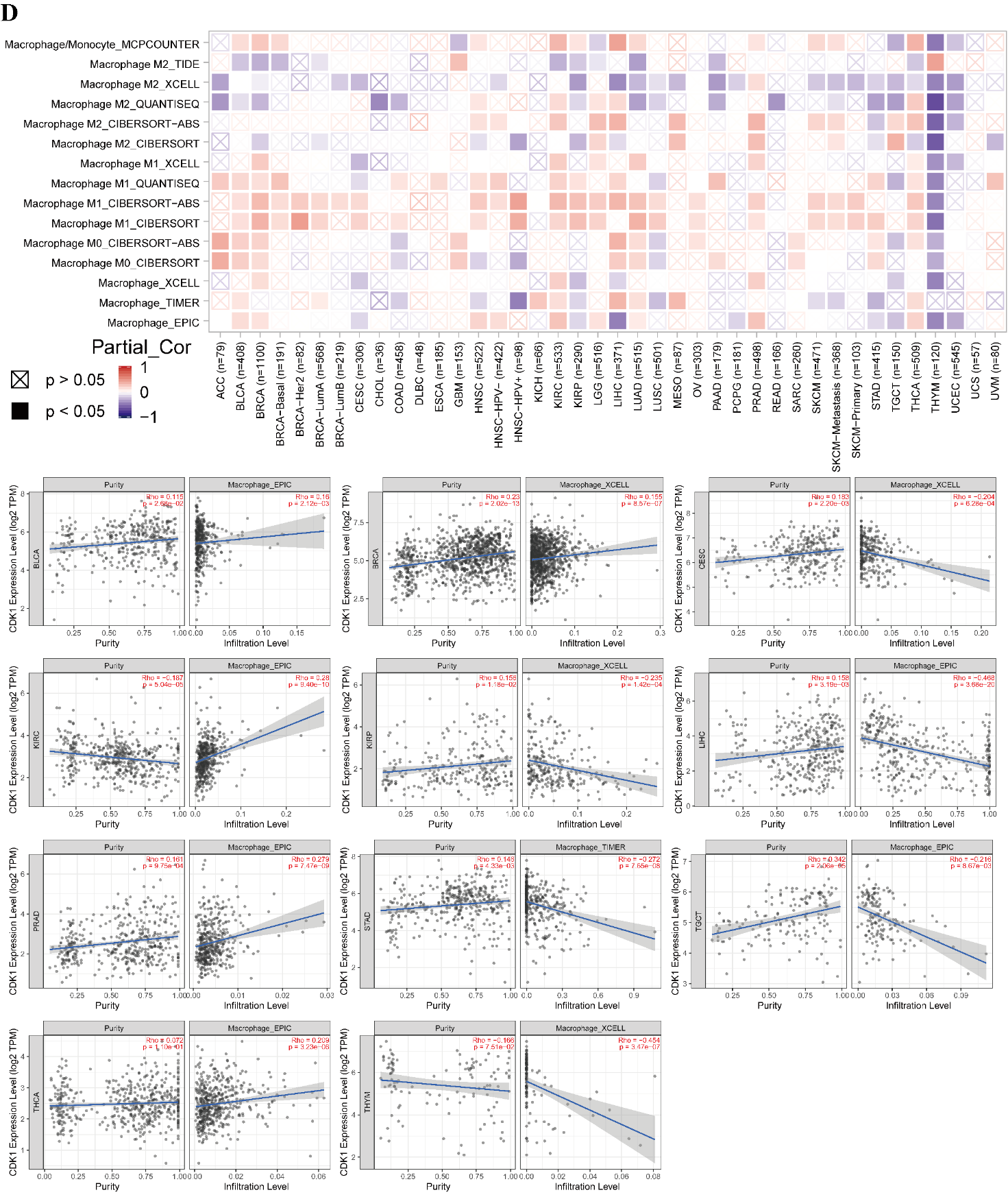

Supplement: Supplementary Materials — Supplementary Table 1. Expression of CDK1 in different types of lung, stomach, liver, colon, breast, esophageal, and pancreatic cancer tissues and normal tissues. Supplementary Figure 1. Kaplan-Meier survival curves comparing the high and low expression of CDK1 in different types of cancer in the (A) PrognoScan database and (B) Kaplan-Meier plotter database. Supplementary Figure 2. Correlation between CDK1 expression in different types of cancer and infiltrating levels of (A) CD8+ T cells, (B) B cells, (C) dendritic cells, and (D) macrophages. [file 8115474.f1.docx]
